# Shiga toxin sub-type 2a increases the efficiency of *Escherichia coli* O157 transmission between animals and restricts epithelial regeneration in bovine enteroids

**DOI:** 10.1371/journal.ppat.1008003

**Published:** 2019-10-03

**Authors:** Stephen F. Fitzgerald, Amy E. Beckett, Javier Palarea-Albaladejo, Sean McAteer, Sharif Shaaban, Jason Morgan, Nur Indah Ahmad, Rachel Young, Neil A. Mabbott, Liam Morrison, James L. Bono, David L. Gally, Tom N. McNeilly

**Affiliations:** 1 Division of Immunity and Infection, The Roslin Institute and R(D)SVS, The University of Edinburgh, Midlothian, United Kingdom; 2 Moredun Research Institute, Penicuik, United Kingdom; 3 Biomathematics and Statistics Scotland, Edinburgh, United Kingdom; 4 Universiti Putra Malaysia, Selangor Darul Ehsan, Malaysia; 5 United States Department of Agriculture, Agricultural Research Service, Nebraska, United States of America; INSERM U1220, FRANCE

## Abstract

Specific *Escherichia coli* isolates lysogenised with prophages that express Shiga toxin (Stx) can be a threat to human health, with cattle being an important natural reservoir. In many countries the most severe pathology is associated with enterohaemorrhagic *E*. *coli* (EHEC) serogroups that express Stx subtype 2a. In the United Kingdom, phage type (PT) 21/28 O157 strains have emerged as the predominant cause of life-threatening EHEC infections and this phage type commonly encodes both Stx2a and Stx2c toxin types. PT21/28 is also epidemiologically linked to super-shedding (>10^3^ cfu/g of faeces) which is significant for inter-animal transmission and human infection as demonstrated using modelling studies. We demonstrate that Stx2a is the main toxin produced by *stx2*a^+^/*stx2c*^*+*^ PT21/28 strains induced with mitomycin C and this is associated with more rapid induction of gene expression from the Stx2a-encoding prophage compared to that from the Stx2c-encoding prophage. Bacterial supernatants containing either Stx2a and/or Stx2c were demonstrated to restrict growth of bovine gastrointestinal organoids with no restriction when toxin production was not induced or prevented by mutation. Isogenic strains that differed in their capacity to produce Stx2a were selected for experimental oral colonisation of calves to assess the significance of Stx2a for both super-shedding and transmission between animals. Restoration of Stx2a expression in a PT21/28 background significantly increased animal-to-animal transmission and the number of sentinel animals that became super-shedders. We propose that while both Stx2a and Stx2c can restrict regeneration of the epithelium, it is the relatively rapid and higher levels of Stx2a induction, compared to Stx2c, that have contributed to the successful emergence of Stx2a+ *E*. *coli* isolates in cattle in the last 40 years. We propose a model in which Stx2a enhances *E*. *coli* O157 colonisation of in-contact animals by restricting regeneration and turnover of the colonised gastrointestinal epithelium.

## Author summary

Enterohaemorrhagic *E*. *coli* (EHEC) O157 strains are found in cattle where they are asymptomatic, while human exposure can lead to severe symptoms including bloody diarrhoea and kidney damage due to the activity of Shiga toxin (Stx). The most serious symptoms in humans are associated with isolates that encode Stx subtype 2a. The advantage of these toxins in the animal reservoir is still not clear, however there is experimental evidence implicating Stx with increased bacterial adherence, immune modulation and suppression of predatory protozoa. In this study, the hypothesis that Stx2a is important for super-shedding and calf-to-calf transmission was tested by comparing excretion and transmission dynamics of *E*. *coli* O157 strains with and without Stx2a. While Stx2a did not alter excretion levels when calfs were orally challenge, it enabled colonisation of more in contact ‘sentinel’ animals in our transmission model. We show that Stx2a is generally induced more rapidly than Stx2c, resulting in increased levels of Stx2a expression. Both Stx2a and Stx2c were able to restrict cellular proliferation of epithelial cells in cultured bovine enteroids. Taken together, we propose that rapid production of Stx2a and its role in establishing *E*. *coli* O157 colonisation in the bovine gastrointestinal tract facilitate effective transmission and have led to its expansion in the cattle *E*. *coli* O157 population.

## Introduction

Enterohaemorrhagic *Escherichia coli* (EHEC) causes life-threatening infections in humans including bloody diarrhoea and kidney failure [1, 2]. O157:H7 is the dominant serotype responsible annually for >15,000 and 1000 human EHEC infections in North America and the United Kingdom respectively [3]. Ruminant livestock, particularly cattle, are the primary reservoir host for EHEC O157:H7 with incidental human infection arising from exposure to contaminated water, meat or vegetables, direct animal and person-to-person contact [4].

The pathogenicity of EHEC O157:H7 is attributed to a Type Three Secretion System (T3SS) encoded by the Locus of Enterocyte Effacement (LEE) pathogenicity island and the production of Shiga toxins (Stx) encoded by lysogenic lambdoid bacteriophage (Φ). Expression of the LEE T3SS is essential for gut colonisation and the formation of attaching and effacing lesions while Stx activity results in the severe life-threatening pathology associated with EHEC infection such as haemorrhagic colitis and haemolytic uremic syndrome (HUS) [1, 5]. Based on protein sequence similarity Stx toxins have been classified into two major groups Stx1 and Stx2, with Stx2 toxins further divided into subtypes Stx2a –g [6, 7]. Disease severity in humans has been strongly correlated with Stx subtype, variant copy number and level of *stx* expression. Strains encoding subtype Stx2a are more likely to cause systemic sequelae [8–10] and this subtype was shown to be a pre-requisite to the development of HUS in a recent analysis of clinical cases in the UK [10]. A recent phylogenomic study has provided evidence that Stx2a was introduced into the UK *E*. *coli* O157 cattle population ~50 years ago, while before this Stx2c had been the main type of Stx2 [10]. The emergence of EHEC O157 as a life-threatening zoonosis is associated with this introduction of the Stx2a subtype. In cytotoxic killing assays Stx2a is 1000 times more toxic to human renal endothelial cells than Stx1 [11, 12]. This toxicity however was significantly reduced in a murine survival model by Stx1 when administered orally with Stx2a, indicating toxin competition for target receptors [13]. Stx toxin genes are encoded in the phage late gene region and are expressed during the phage (Φ) lytic cycle that culminates in host cell lysis and toxin release. Expression can be induced using DNA damaging agents such as mitomycin C and requires the phage-encoded anti-terminators Cro and N (early gene expression) and Q (late gene expression). Induced *stx2a* has been shown to be expressed at significantly higher levels than *stx2c* or other *stx2* variants from single Stx2a-encoding prophage (Φstx2a) or double Φstx2a Φstx2c lysogens [14–16]. Ogura *et al* (2015) characterised 6 subtypes of Φstx2a according to their replication proteins each producing distinct levels of Stx2a. Importantly strains conferring the highest Stx2a production clustered with hyper-virulent clade 8 strains [17].

In cattle, colonisation by EHEC O157:H7 is considered asymptomatic and whether and how Stx may confer a selective advantage in cattle is still not clear. Stx has been shown in cell culture and mice to alter the expression and localisation of receptors for the bacteria that include nucleolin and integrin [18, 19], and these can facilitate *E*. *coli* O157 colonisation; however evidence in cattle is lacking. Stx1 and Stx2 have been shown to suppress host innate and adaptive immune responses during colonisation although whether this leads to increased bacterial excretion or promotes colonisation has not been demonstrated [18, 20–24]. It has also been proposed that Stx may be important for killing of grazing protozoa that predate on *E*. *coli* in the rumen, although there is conflicting data to support this interesting ecological concept [25, 26]. Importantly certain Stx subtypes, in particular Stx2a, have an epidemiological association with increased excretion levels of *E*. *coli* O157 from cattle, also known as super-shedding [27–30]. Cattle excreting *E*. *coli* O157:H7 at levels > 10^3^ cfu/g faeces have been termed super-shedders and significantly affect prevalence levels on-farm and transmission probabilities between animals [31, 32]. In a recent analysis of faecal pat and clinical isolates of *E*. *coli* O157 collected in Scotland from 2002–2004, Matthews *et al* (2013) deduced that the Stx2a variant, alone or in combination with Stx2c, was a critical factor for super-shedding and that it increased the risk of human infection. Previous prevalence studies have estimated that 9–20% of animals within an *E*. *coli* O157 positive herd are super-shedders, however these few high shedding animals can account for > 90% of the total *E*. *coli* O157 present in feed-lot pens and are predicted to account for 80% of animal-to-animal transmission [32–38].

In the UK, *E*. *coli* O157 strains are subtyped according to their sensitivity to a panel of 16 bacteriophages [10]. Over the last decade Lineage I (Lineage Ic) strains of phage type (PT) 21/28 have been associated with both severe human disease and super-shedding in cattle, particularly in Scotland which has one of the highest rates of EHEC O157 incidence in the world [10, 32, 39, 40]. Analyses of faecal pat prevalence data on Scottish farms from two major surveys (SEERAD 1998–2000, IPRAVE 2002–2004) determined PT21/28 strains were more likely to be associated with super-shedding, a higher rate of animal-to-animal transmission and an increased risk of severe disease in humans compared with PT32 strains, a PT found in 10% of Scottish cattle [27, 36, 38]. Critically, toxin subtype Stx2a was encoded by the majority of PT21/28 strains, either alone or in combination with Stx2c, compared with the Scottish PT32 cattle strains which tended to encode stx2c only [28].

A recent phylogenetic study of *E*. *coli* O157 in the UK concluded that PT32 was a direct ancestor of PT21/28 strains [10] and in fact integration of Φstx2a into a PT32 Φstx2c background can result in PT conversion from PT32 to PT21/28 [41]. PT21/28 has therefore evolved from PT32 in part by acquisition of Φstx2a. In the present study we have set out to investigate the biology of Stx2a in PT21/28 strains, including, (1) understanding toxin expression when both Stx2a and Stx2c can be produced; (2) testing directly the impact of Stx2a on both the super-shedding phenotype and transmission between animals. We provide evidence that while both Stx2a & 2c can inhibit epithelial cell regeneration, Stx2a is induced to higher levels and more rapidly than Stx2c in PT21/28 strains. In a series of controlled cattle trials we show that calves orally dosed with a PT21/28 strain shed at significantly higher levels than those dosed with a PT32 strain. In transmission experiments, functional Stx2a was shown to be important for establishing a super-shedding phenotype in sentinel calves. We propose a model for how Stx2a and its carrier prophage combine in *E*. *coli* O157 PT21/28 strains to promote colonisation and transmission in cattle.

## Results

### Stx2a-producing PT21/28 strains produce more Stx2 than strains of other phage types producing Stx2c alone

**Fig 1:**
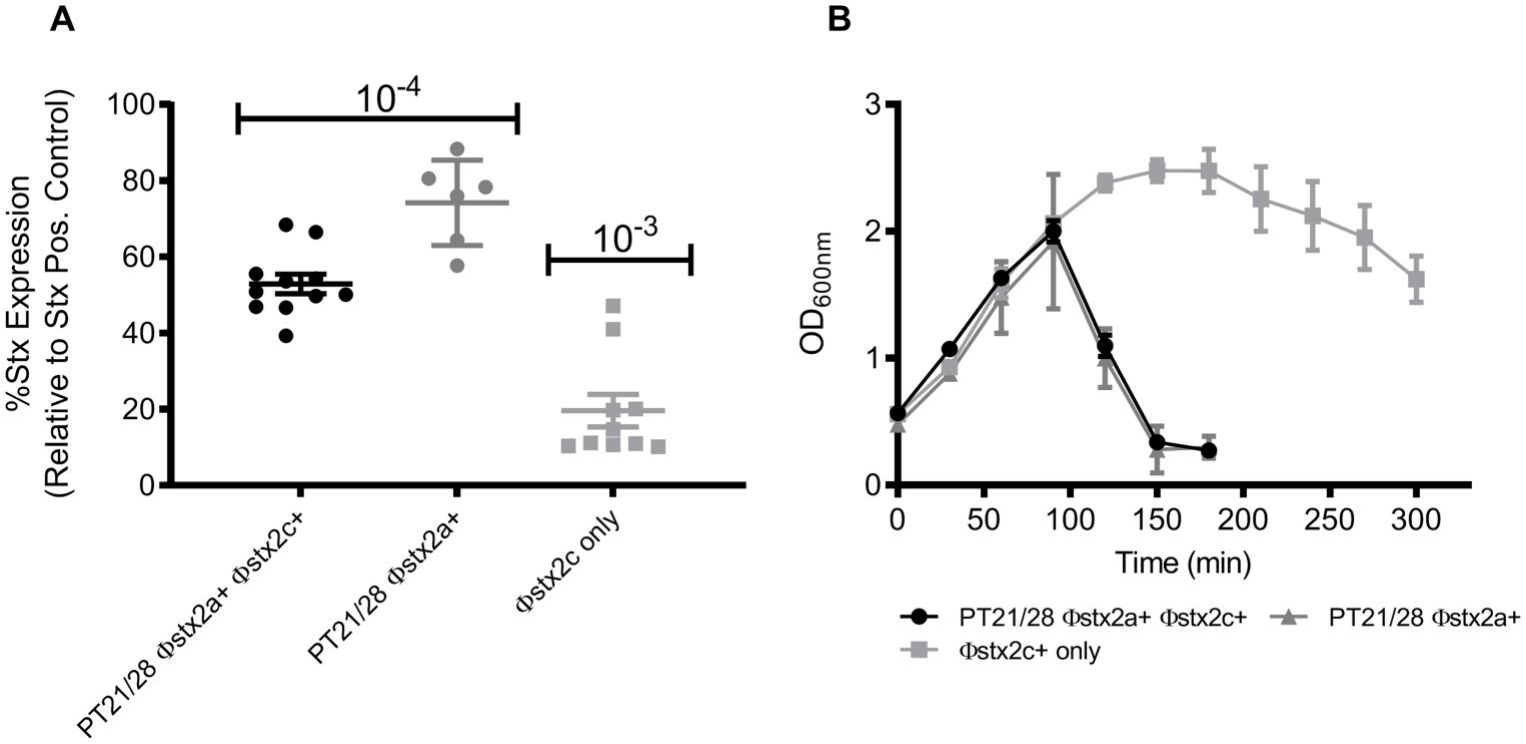
Comparison of Stx2 toxin levels and lysis for PT21/28 Φstx2a^+^ and Φstx2c encoding strains. (A) Total Stx2 toxin produced by PT21/28 Φstx2a^+^ Φstx2c^+^ (n = 10), PT21/28 Φstx2a^+^ (n = 6) and Φstx2c (n = 10) carrying strains measured by ELISA assay after 24 hrs induction with MMC. Values are expressed relative to a Stx positive control used in each assay. The individual strain values and mean ± SEM are shown. 10^−4^ and 10^−3^ indicate the dilution factor for the samples used for the Stx2 toxin ELISA assay. (B) Lysis of PT21/28 Φstx2a^+^ Φstx2c^+^ (n = 10) (black circles), PT21/28 Φstx2a^+^ (n = 6) (grey triangle) and Φstx2c (n = 10) (grey square) encoding strains was measured spectrophotometrically as a drop in culture OD_600nm_. The mean OD_600nm_ values ± SEM are plotted.

Epidemiological studies provide evidence that PT21/28 Stx2a+ strains are associated with super-shedding and serious disease in humans compared to other PTs producing Stx2c only [10, 28]. We wanted to determine if carrying ΦStx2a leads to increased Stx production compared to ΦStx2c. Stx toxin levels of sixteen PT21/28 Φstx2a^+^ isolates were compared with ten isolates encoding only Φstx2c (details of the strains used are provided in Table 1). The Stx type of all selected isolates was determined previously by PCR or illumina sequencing [28, 42, 43]. Selected PT21/28 isolates encoded either Φstx2a alone or in combination with Φstx2c. Isolates encoding only Φstx2c comprised a mixture of PT32 (n = 7), PT34 (n = 2) and PT49 (n = 1). As determined using a commercial Stx2 ELISA, the PT21/28 Φstx2a^+^ isolates produced significantly higher (p < 0.0001) levels of Stx2 toxin compared with isolates carrying only Φstx2c (Fig 1A). To be within the limits of detection for the toxin assay, samples from Φstx2a^+^ isolates were diluted 1 x 10^−4^ while Φstx2c only samples were used at 1 x 10^−3^; therefore PT21/28 isolates encoding Φstx2a produced >10-fold more Stx2 than strains with just Φstx2c. There was no evidence of a cumulative effect of possessing both Φstx2a and Φstx2c phage, in fact PT21/28 isolates with Φstx2a alone produced significantly (p = 0.0019) more Stx2 than isolates with both phage (Fig 1A).

**Table 1:** Details of bacterial strains used in this study.

Strain	Shiga Toxin gene (s)	Phage Type	Origin	Modification	Source/Reference
9000	*stx2a*, *stx2c*	PT21/28	Cattle faeces	None	[40]
10671^a^	*stx2c*	PT32	Cattle faeces	None	[40]
9000R	*stx2a*, *stx2c*	PT21/28	Strain 9000	ΔISEc8 stx2a	This study
ZAP1380	*stx2a*, *stx2c*	PT21/28	Strain 9000	Nal^R^	[76]
ZAP1381	*stx2c*	PT32	Strain 10671	Nal^R^	[76]
ZAP1723	*stx2a*, *stx2c*	PT21/28	Strain 9000R	Nal^R^	This study
ZAP1460	*stx2c*	PT32	Strain 9000	ΔΦstx2a	[41]
ZAP1452	*stx2a*	PT21/28	Strain 9000	ΔΦstx2c	[41]
ZAP1463	None	NT	Strain 10671	ΔΦstx2c	[41]
ZAP1465	None	NT	Strain 9000	ΔΦstx2a/ΔΦstx2c	[41]
ZAP563^a^	*stx2a*, *stx2c*	PT21/28	Cattle faeces	None	[40]
ZAP564^a^	*stx2a*	PT21/28	Cattle faeces	None	[40]
ZAP859^a^	*stx2a*, *stx2c*	PT21/28	Cattle faeces	None	[40]
ZAP882^a^	*stx2a*, *stx2c*	PT21/28	Cattle faeces	None	[40]
ZAP885^a^	*stx2a*, *stx2c*	PT21/28	Cattle faeces	None	[40]
ZAP903^a^	*stx2a*, *stx2c*	PT21/28	Cattle faeces	None	[40]
ZAP909^a^	*stx2a*, *stx2c*	PT21/28	Cattle faeces	None	[40]
ZAP1478^a^	*stx2a*, *stx2c*	PT21/28	Cattle faeces	None	[40]
ZAP1504^a^	*stx2a*, *stx2c*	PT21/28	Cattle faeces	None	[40]
ZAP1625^a^^,^^b^	*stx2a*, *stx2c*	PT21/28	Human	None	[40]
ZAP1626^a^^,^^b^	*stx2a*, *stx2c*	PT21/28	Human	None	[40]
ZAP1634^a^^,^^c^	*stx2a*	PT21/28	Human	None	
ZAP1635^a^^,^^c^	*stx2a*	PT21/28	Human	None	
ZAP1823^a^^,^^b^	*stx2a*	PT21/28	Human	None	
ZAP1824^a^^,^^b^	*stx2a*	PT21/28	Bovine	None	
ZAP1831^a^^,^^b^	*stx2a*	PT21/28	Bovine	None	
ZAP858^a^	*stx2c*	PT32	Cattle faeces	None	[40]
ZAP875^a^	*stx2c*	PT32	Cattle faeces	None	[40]
ZAP877^a^	*stx2c*	PT32	Cattle faeces	None	[40]
ZAP881^a^	*stx2c*	PT32	Cattle faeces	None	[40]
ZAP884^a^	*stx2c*	PT32	Cattle faeces	None	[40]
ZAP895^a^	*stx2c*	PT32	Cattle faeces	None	[40]
ZAP1493^a^	*stx2c*	PT34	Cattle faeces	None	[40]
ZAP1494^a^	*stx2c*	PT34	Cattle faeces	None	[40]
ZAP1549^a^	*stx2c*	PT49	Cattle faeces	None	[40]

^a^Isolate used for PT21/28 and PT32 lysis and toxin comparisons;

^b^Strain provided by Scottish *E*. *coli* Reference Laboratory (SERL);

^c^Strain provided by Public Health England (PHE) NT = not tested; Nal^R^ = nalidixic acid resistant.

The best characterised route to Stx2 release is phage-mediated lysis of the host bacterium. To examine if lysis kinetics were distinct for isolates containing the different Stx2-encoding prophages, the timing of lysis following induction with mitomycin C (MMC, 2 μg/ml) was determined for each isolate. All PT21/28 isolates with Φstx2a had a rapid lysis phenotype compared to those with only Φstx2c (Fig 1B). Lysis began after 90 min and complete lysis was observed for every strain tested at 180–210 min post-induction. Lysis of isolates with only Φstx2c was more variable but in general all strains had a similar lysis phenotype with cessation of growth occurring after 120 min followed by a gradual decline in culture OD up to 300 min (Fig 1B). Complete lysis was observed for all strains after 24 h. These results indicate that Φstx2a have a rapid lysis phenotype when induced and produce more Stx2 than Φstx2c alone in the phage types tested.

#### Rapid activation of Φstx2a gene expression leads to dominant Stx2a production

The above results indicate that Φstx2a may be the main source of Stx2 toxin in strains with both Stx2a and Stx2c prophages. To investigate this further, we selected and generated a series of strains which vary in their Stx phage repertoire and capacity to produce Stx2a. As we intended to compare excretion and transmission of strains with and without Stx2a, we chose strains isolated from cattle as part of a national survey [40]. PT21/28 strain 9000 was associated with a high single pat count (6.9 x 10^5^ cfu/g) and was positive for both *stx2a* and *stx2c* toxin gene variants by PCR and PT32 strain 10671 which was only detected by enrichment in a faecal pat (<50 cfu/g) and was positive for only *stx2c* by PCR. Following long-read sequencing of strain 9000 we identified that the *stx2A* subunit gene contained an ISEc8 insertion sequence (IS) element at position +594 relative to ATG (S1 Fig). This strain therefore served as a natural Stx2a null mutant. We precisely removed the ISEc8 from the *stx2A* gene by allelic replacement to generate a ‘repaired’ 9000 variant termed 9000R. These three strains: 9000 (*stx2a*::ISEc8 and *stx2c*^*+*^), 9000R (ΔISEc8 *stx2a*^*+*^ and *stx2c*^*+*^) and 10671 (*stx2c*^+^) along with isogenic derivatives in which either Stx bacteriophage Φstx2a or Φstx2c or respective toxin genes, *stx2a* or *stx2c*, were deleted (Table 1) were compared for Stx2 toxin production, lysis and Φstx2a/c gene expression.

**Fig 2:**
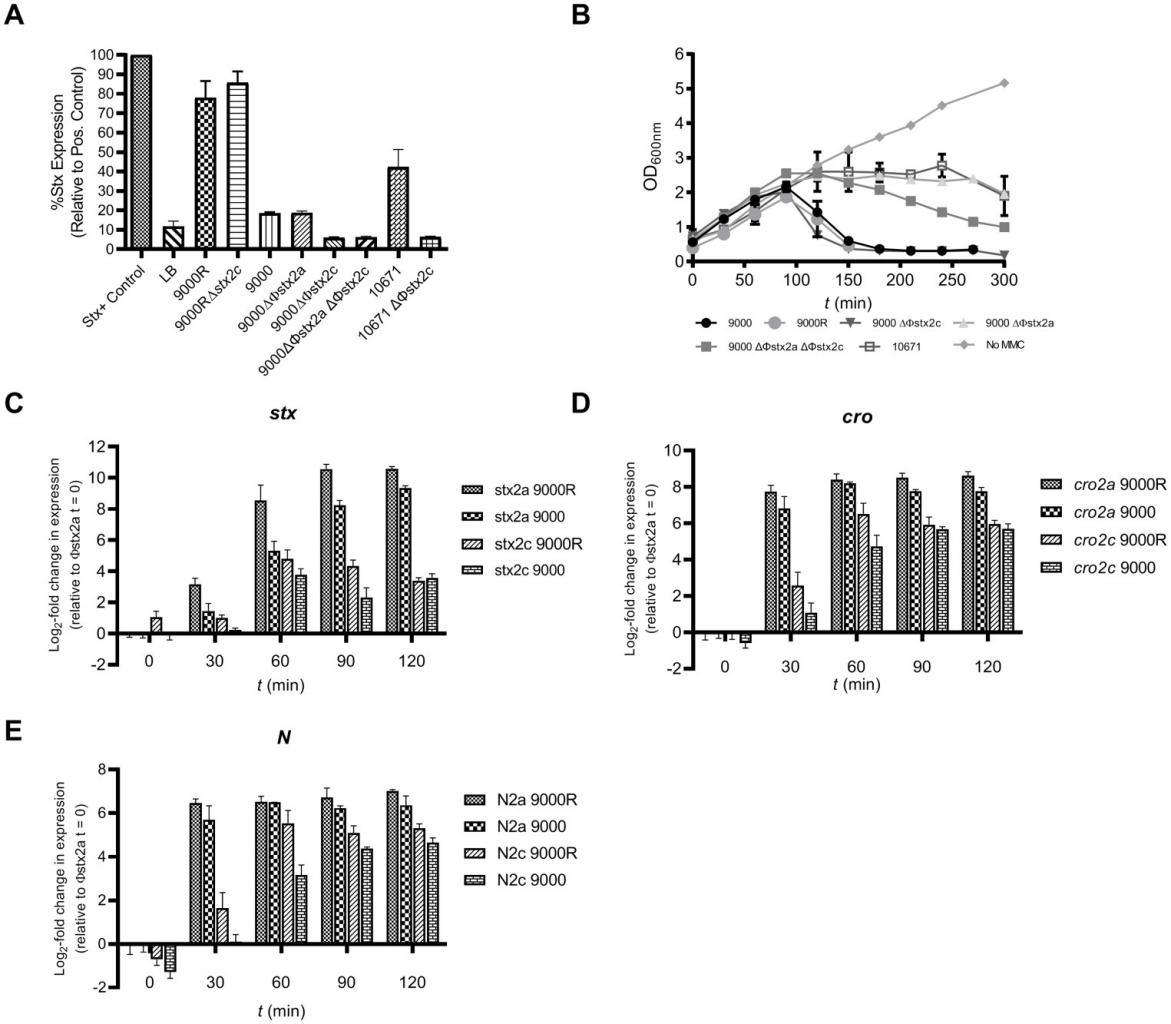
Stx2 toxin production, lysis and Φstx2 gene expression. (A) Pan Stx2 toxin production from PT21/28 strains 9000, 9000R, PT32 strain 10671 and genetic derivatives was measured by ELISA 24 h post induction with MMC. Values are expressed relative to an inactivated Stx toxin control used in each assay. The mean ± SEM from four biological replicates (n = 4) are shown for each strain. Samples were first diluted either 10^−4^ or 10^−3^ to be within the assay limits of detection. LB = Lysogeny Broth without bacteria. (B) Lysis of strains 9000, 9000R, 10671 and genetic derivatives was measured spectrophotometrically as a drop in culture OD_600nm_. Mean OD_600nm_ values ± SEM from four biological replicates (n = 4) are shown. No MMC = growth of strain 9000 without MMC. Expression of the late gene *stx2B* (C) and early gene regulators *cro* (D) and *N* (E) was monitored from both Φstx2a and Φstx2c in strains 9000 and 9000R after induction with MMC. The mean ± SEM from four biological replicates (n = 4) is shown. Data for *stx2*, *N* and *cro* genes is expressed relative to the Φstx2a encoded analogues (*stx2a*, *N2a*, *cro2a*) at time (*t)* = 0, respectively.

Upon removal of ISEc8 from the Stx2a A subunit gene in strain 9000R, pan-Stx2 toxin production was now >10-fold higher than strains 10671 or 9000, confirming that the ISEc8 had prevented Stx2a production in strain 9000 (Fig 2A). Comparable toxin levels were observed for both 9000R and 9000R Δ*stx2c* indicating that repair of *stx2a* was responsible for the significant increase in toxin levels relative to 9000 and 10671 and that Stx2a is the primary toxin produced by 9000R (Fig 2A). The ΔΦstx2a 9000 derivative produced equivalent Stx2 levels to 9000 confirming that all toxin produced by the WT strain 9000 was Stx2c and that *stx2a* was inactivated by ISEc8. The strain 9000 ΔΦstx2a ΔΦstx2c double deletion produced no Stx compared to a Lysogeny broth (LB) control as was the case for strain 10671 ΔΦstx2c (Fig 2A).

Lysis of wildtype strains 9000 (Φstx2a^+^ Φstx2c^+^), 9000R (Φstx2a^+^ Φstx2c^+^), 10671 (Φstx2c^+^) and isogenic derivatives was monitored after induction with MMC (2 μg/ml) (Fig 2B). Without induction (no MMC) all strains grew comparably. When induced lysis of strains 9000 and 9000R began after 90 min and complete lysis occurred 180–210 min post-induction. Φstx2c did not influence this lysis phenotype as strain 9000 ΔΦ*stx2c* had an equivalent lysis curve. In contrast when Φstx2a was deleted lysis of strain 9000 ΔΦstx2a was comparable to strain 10671 which carries Φstx2c^+^ but not Φstx2a^+^. Growth of both strains stopped at 120 mins post-induction but no cellular lysis occurred as no drop in culture OD was recorded for the duration of the experiment (Fig 2B). Lysis of a double Φstx2 phage deletion strain, 9000 ΔΦstx2a ΔΦstx2c, was also monitored which revealed a slow lysis phenotype. Lysis of strain 9000 ΔΦstx2a ΔΦstx2c began after 90 min post-induction and proceeded at steady rate thereafter (-0.23 OD_600nm_ /30 min). It should be noted that complete lysis of strains 9000 ΔΦstx2a, 9000 ΔΦstx2a ΔΦstx2c and 10671 (Φstx2c^+^) was observed after overnight incubation for 24 h.

As demonstrated in Fig 2A, Stx2 levels produced from Φstx2a in PT21/28 strain 9000R were significantly higher than levels of Stx2c produced from Φstx2c. To test if this higher level of toxin production results from increased *stx2a* expression relative to *stx2c*, expression of the *stx2* B subunit gene was measured in strains 9000 and 9000R after induction of phage replication (MMC, 2 μg/ml) (Fig 2C). Expression of the early gene regulators *cro* (Fig 2D) and *N* (Fig 2E) were also determined from Φstx2a (*cro2a*, *N2a*) and Φstx2c (*cro2c*, *N2c*). At 30 min post-induction the expression of *N2a* and *cro2a* in both 9000 and 9000R, increased 6–8-fold and expression of *stx2a*, encoded within the late gene region, increased 2–4-fold. In contrast, basal levels of expression of *stx2c*, *N2c* and *cro2c* (0.2–2.5-fold change) were observed at this same time point. A plateau in the expression of both Φstx2a- and Φstx2c-located genes occurred 60–90 min post-induction however all Φstx2a-located genes (*stx2a*, *N2a* and *cro2a*) were expressed at significantly higher levels (*p* < 0.05) than their Φstx2c-encoded analogues at every time point in both strain 9000 and strain 9000R. As *cro* and *N* control early phage gene expression during replication these results indicate that Φstx2a initiates replication faster than Φstx2c and once started significantly more Stx2a is produced. Consequentially the strains carrying Φstx2a produce higher levels of Stx2 toxin relative to Φstx2c.

### Stx2a and Stx2c inhibit budding of bovine ileal organoids

The established receptor for Stx subtypes is Gb3/CD77 [44, 45]. We have previously shown that the Stx toxin receptor, Gb3, is expressed within bovine ileal crypts and demonstrated Stx1 toxin binding in the basal region of isolated crypts [46, 47]. We hypothesized that Stx2a/c may also target these cells and impact on epithelial function and homeostasis to the advantage of the bacterium. Therefore, any differences in Stx2 production by *E*. *coli* O157 strains *in vivo* may lead to differential effects on such a phenotype. To investigate the activities of Stx2-containing supernatants, the budding phenotype of bovine ileal organoids (‘miniguts’) was assessed. These organoids consist of a central internal lumen lined with a single layer of polarised enterocytes including intestinal stem cells analogous to those within ileal crypts, and form new ‘buds’ as a result of intestinal stem cell proliferation [48].

**Fig 3:**
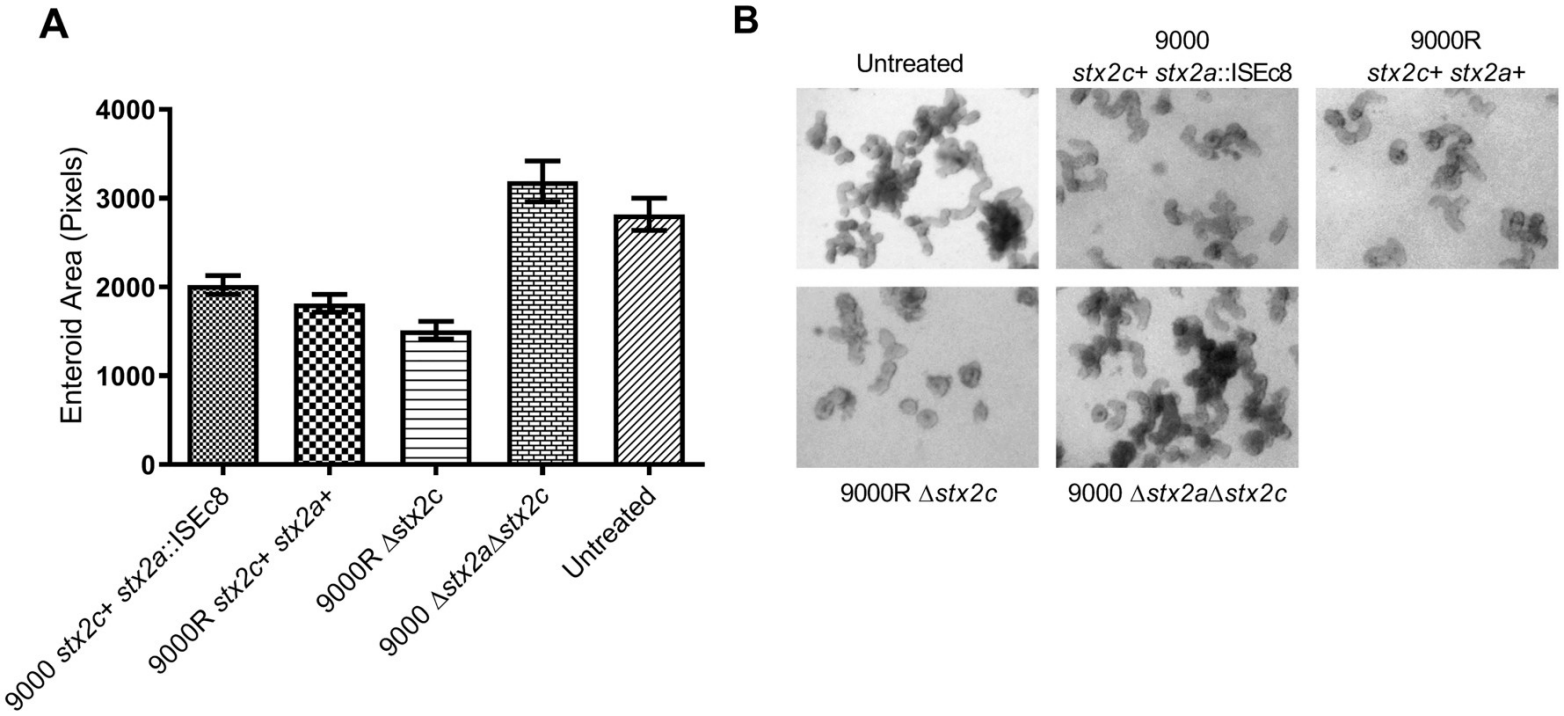
Stx2 inhibition of ileal organoid budding. Mean organoid size (A) and representative images of Stx2 treated or untreated organoids (B) are shown. Organoids were treated with supernatants derived from strains 9000 (n = 3784), 9000R (n = 4005), 9000R Δ*stx2c* (n = 964), 9000 Δ*stx2a*Δ*stx2c* (n = 1540) or untreated (n = 1409). Organoid size was determined from organoids treated with a range of toxin supernatant dilutions (1/200–1/500) used in each experimental replicate. Mean organoid size ± SEM is shown for four experimental replicates (n = 4).

Organoid cultures were treated with toxin-containing supernatants derived from strain 9000 (*stx2c*+ *stx2a*::ISEc8) and 9000R (*stx2c*+ *stx2a*+) after induction with MMC and organoid size/budding was quantified after 7-days (Fig 3). Treatments occurred during passage of the organoids, when mechanical disruption of organoid exposes the internal luminal surface. Toxin-containing supernatants from both 9000 and 9000R significantly inhibited organoid budding (*p* < 0.0001) compared to untreated control organoids (Fig 3). There was evidence that supernatants from strain 9000R (*stx2c*+ *stx2a*+) inhibited budding to a greater extent than organoids treated with supernatants from strain 9000 (*stx2c*+ *stx2a*::ISEc8) (*p* = 0.0001). Similarly, inhibition of budding by supernatants containing Stx2a only (9000R Δ*stx2c*) was comparable to 9000R. All inhibition of budding was toxin dependent as treatment with supernatants derived from a toxin negative control strain, 9000 Δ*stx2a*Δ*stx2c*, did not inhibit budding relative to untreated organoids. To test if treatment with Stx induced apoptosis/cell death, organoids treated with toxin-containing supernatants from strains 9000, 9000R and untreated control organoids were stained for dead cells after 7-days (S2 Fig). In both control and Stx treated organoids dead cells were found to accumulate within the organoid lumen, with no evidence of any dead cells in the periphery of either treated or untreated organoids. Furthermore upon passage without toxin, budding of Stx treated organoids was recovered to levels (~72–74% budding) comparable with untreated controls. These results indicate that both Stx2a and Stx2c can restrict normal cellular proliferation within bovine crypts independent of cell death/apoptosis with evidence of increased activity in the presence of Stx2a.

### Increased shedding and animal-to-animal transmission associated with functional Stx2a

To assess the contribution that acquisition of Φstx2a by PT21/28 strains had on ‘super shedding’ and animal-to-animal transmission, three separate animal trials were conducted in which calves were challenged with Nal^R^ derivative strains of PT32 10671 (trial 1), PT21/28 9000 (trial 2) or PT21/28 9000R (trial 3). For each trial animals were housed in each of three rooms (C1, C2 and C3) as detailed in S3 Fig. On day 0 all animals in C1 were orally challenged with ≈1x10^9^ cfu/ml of the inoculum strain and bacterial shedding was monitored by faecal counts (cfu/g) for all challenged animals over a 25-day period (S4 Fig). On Day 5, when peak shedding was observed, one high shedding challenged animal was moved into C2 and another into C3, each room housing five naïve sentinel calves. All transmission events between challenged and sentinel animals were monitored by faecal counts (cfu/g) over an 18-day period (S4 Fig). Environmental levels of each challenge strain were also monitored in rooms C1 –C3 for the duration of each trial. It is noteworthy that environmental levels mirrored that of the highest shedding animal in each room (S4 Fig) indicating that the environment did not act as a significant reservoir of infection under these experimental conditions.

#### Shedding analysis for challenged animals

**Fig 4:**
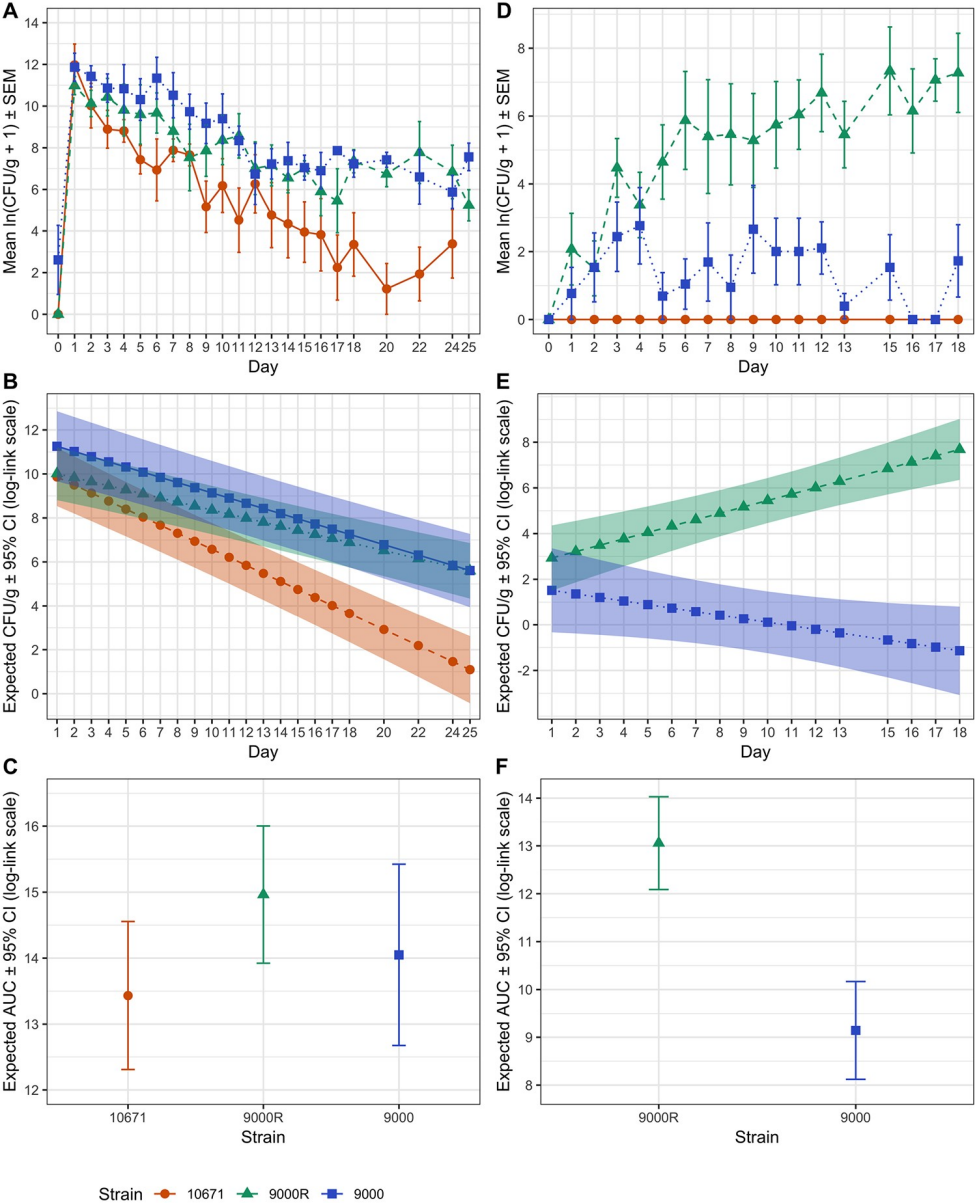
Analysis of shedding and transmission from experimentally challenged and sentinel calves. Shedding data and analysis is shown for experimentally infected calves (A–C) and naïve in-contact sentinel calves (D–F). (A) Mean ± SEM daily cfu/g faeces from animals challenged with strains 10671 (n = 4) (red), 9000R (n = 7) (green) and 9000 (n = 6) (blue). (B) Strain specific differences in mean cfu/g over time were determined using a Poisson GLMM. Predicted mean cfu/g values ± 95% confidence interval (CI) bands (in log-link scale) are shown for 10671 (red), 9000R (green) and 9000 (blue). (C) Predicted total shedding for each strain was calculated by AUC analysis. Mean AUC ± 95% CI (in log-link scale) are shown. Equivalent analysis for all sentinel animals infected with strains 10671, 9000R and 9000 by transmission was performed. (D) Mean daily ± SEM cfu/g faeces (in log scale), (E) GLMM predicted mean daily cfu/g ± 95% CI bands (in log-link scale) and (F) negative binomial GLM predicted mean AUC ± 95% CI (in log-link scale) for sentinel animals are shown.

Faecal samples from animals challenged with strains 10671 (n = 6), 9000 (n = 4) and 9000R (n = 7) were collected daily for 18-days post challenge and on alternate days thereafter and the mean daily cfu/g faeces enumerated from triplicate plate counts (Fig 4A). Mean cfu/g counts over time were modelled using a Poisson generalised linear mixed model (GLMM) to determine strain specific differences. Statistically significant differences in mean cfu/g over time were observed between strains with mean counts for PT32 strain 10671 predicted to diverge from strains 9000 and 9000R (Fig 4B). Pairwise testing of the differences in mean cfu/g between strains further confirmed that calves challenged with PT32 strain 10671 had significantly lower daily mean bacterial shedding compared with calves challenged with either PT21/28 strain 9000 or 9000R (p = 0.012 and p = 0.018, respectively). No statistically significant differences in shedding between strain 9000 and 9000R was observed (p = 0.454). Total bacterial excretion over the duration of the trial was calculated by Area under the Curve (AUC) analysis and a negative binomial GLM was used for strain comparison. No statistically significant differences in mean were observed between the three strains (p = 0.108) (Fig 4C). Thus in our high dose oral challenge model the ability to produce active Stx2a toxin had no significant effect on shedding as strains 9000 and 9000R had equivalent predicted mean shedding curves.

#### Animal-to-animal transmission

Transmission of strains 10671, 9000 and 9000R between experimentally infected and sentinel animals was monitored by enumerating cfu/g faeces from all sentinel animals in rooms C2 (n = 5) and C3 (n = 5) (S4 Fig). The mean daily cfu/g faeces from animals colonized by each strain over the 18-day trial period are plotted in Fig 4D. Both PT21/28 strains 9000 and 9000R were transmitted to 9/10 and 10/10 sentinel animals, respectively. Furthermore 6/10 animals colonised by strain 9000R became super-shedders, many of which excreted > 1 x 10^3^ cfu/g for several consecutive days (S4 Fig). In contrast detection of strain 9000 was sporadic and generally required sample enrichment with the exception of one super-shedding event lasting just 2-days (S4 Fig). PT32 strain 10671 did not transmit to any sentinel animals and so was excluded from any further analysis. As above, statistical modelling was used to determine strain specific differences between strains 9000 and 9000R. Significantly higher mean cfu/g counts over time were predicted for strain 9000R compared to stain 9000 (*p* < 0.001, Fig 4E) with the difference in mean cfu/g counts between 9000R and 9000 predicted to increase 0.43 log cfu/g per day. The mean total cfu/g faeces (AUC) for strain 9000R was also estimated to be ~ 50 fold greater than strain 9000 (*p* < 0.001) (Fig 4F).

#### Stx2a does not suppress immune recognition

Stx2 was previously shown to suppress adaptive immune responses in cattle [22]. We hypothesized that Stx2a mediated suppression of *E*. *coli* O157-specific adaptive immune responses by 9000R within the intestinal mucosa may increase colonisation of the bovine intestinal tract and account for the enhanced transmission phenotype. To test this we characterised the circulating and mucosal *E*. *coli* O157-specific antibody responses in calves orally challenged with 9000, 9000R and 10671 during the above challenge trials.

**Fig 5:**
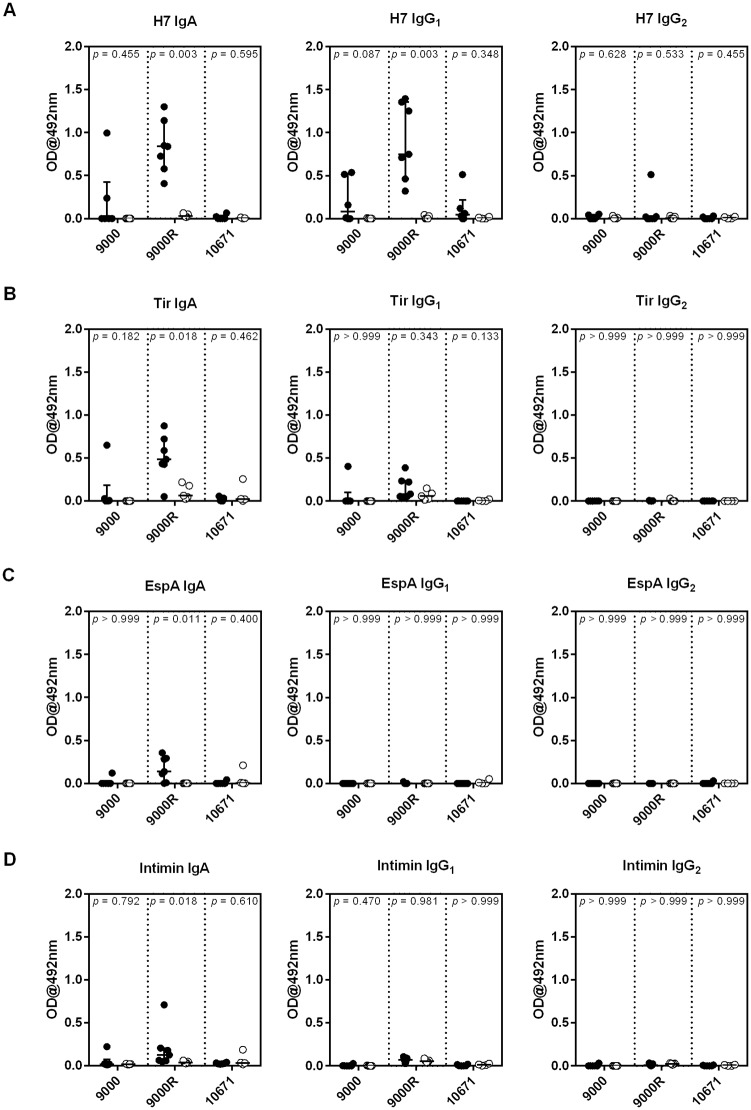
Enhanced rectal antibody responses to *E*. *coli* O157 antigens following challenge with a Stx2a^+^ *E*. *coli* O157 strain. Antibody Secreting Cell (ASC) probes were generated from rectal lymph node cells isolated at post-mortem from calves orally challenged with ~10^9^ cfu of *E*. *coli* O157 strains 9000, 9000R, and 10671 and from unchallenged control calves. Levels of (A) H7-specific; (B) Tir-specific; (C) EspA-specific and (D) Intimin-specific IgA, IgG_1_ and IgG_2_ within ASC probes quantified by ELISA. Each symbol represents an individual animal and medians and interquartile ranges are presented. Mann Whitney *U-*tests were used to compare antibody levels between challenged and unchallenged controls for each *E*. *coli* O157 strain and associated *p*-values are indicated.

Weekly serum antibody responses to four *E*. *coli* O157 antigens: H7, EspA, Intimin and Tir, involved in protective immunity to *E*. *coli* O157 [49, 50], are shown in S5 Fig. Challenge with all three strains induced a significant increase in serum H7-specific IgA (*p* < 0.01). An increase in serum levels of H7-specific IgG_1_ was also seen in calves challenged with strain 9000R only (*p* = 0.029). A small but significant increase in Tir and EspA-specific IgG1 was also observed in calves challenged with strain 10671 (*p* = 0.045 & 0.023 for Tir and EspA, respectively) but not the 9000 or 9000R strains. As the predominant colonisation site of *E*. *coli* O157 in cattle is the terminal rectum [51], Antibody Secreting Cell (ASC) probes [52] were generated from rectal lymph nodes collected at post-mortem from challenge and unchallenged control calves to quantify antibody responses local to the site of colonisation. ASC probes from calves challenged with strain 9000R had significantly higher levels of H7, Tir, EspA and Intimin-specific IgA (*p* = 0.003, 0.018, 0.011 and 0.018, respectively), and H7-specific IgG_1_ (*p* = 0.003) compared to their unchallenged controls, whereas challenge with strains 9000 and 10671 resulted in no detectable increase in antibodies to any of the four *E*. *coli* O157 antigens tested (Fig 5). This indicates that local *E*. *coli* O157-specific antibody responses were greatest following challenge with strain 9000R and Stx2a-enhanced transmission of the PT21/28 strain is unlikely to be due to suppression of *E*. *coli* O157-specific immune responses at the site of colonisation.

## Discussion

Within different geographical regions such as North and South America, the UK and Sweden, different *E*. *coli* O157 subtypes are present in the cattle population that encode the Stx2a subtype and these are associated with serious pathology in humans [40, 53, 54]. Stx2a has emerged in the last 50 years in the cattle population with the prophage inserting into strain backgrounds often already containing Stx2c [10, 55]. In the UK, this has led to the emergence of Stx2a^+^2c^+^ PT21/28 isolates which have been the most significant O157 subtype associated with life-threatening human infections in the UK over the last 15 years [10]. In the current study, our analysis of phage induction kinetics and toxin production in *E*. *coli* O157 PT21/28 backgrounds demonstrated that Stx2a-encoding prophage are generally induced more rapidly than those encoding Stx2c. The more rapid induction and lysis kinetics means that Stx2a becomes the dominant Stx2 subtype produced by these isolate populations, thus reducing any selection pressure that would have been applied by production of Stx2c. Studies have shown that Stx2c phage (Φ) have undergone significant gene loss over time particularly in genes required for lysis, replication and repair [41, 56]. We also identified an IS element inserted in the excisionase (xis) gene of ΦStx2c in strain 9000 that prevented precise excision [41]. Such targeted gene loss and IS interruptions preventing precise excision may account for the observed slow lysis phenotype in the absence of ΦStx2a. In contrast phage producing Stx2a are diverse and six ΦStx2a sub-types have recently been classified based upon differences within phage regulatory regions which, in part, correlate with different levels of toxin production [16, 17, 41].

Based on our findings, it is likely that Stx2a is also the main toxin subtype produced by PT21/28 strains following induction in the bovine host. There is an epidemiological association between encoding *stx2a* and higher levels of excretion from cattle (super-shedding)[27–30] and a primary aim of the current study was to experimentally investigate the role of Stx2a in shedding and transmission dynamics of *E*. *coli* O157 within the primary bovine reservoir. A key finding was that restoring the capacity to produce Stx2a in PT21/28 strain 9000 significantly increased excretion from sentinel calves co-housed with a shedding animal. Despite the fact that such experiments were logistically difficult to arrange under restrictions in the UK on working with Stx^+^ isolates, a successful protocol was established in which experimentally-colonized Trojan calves were introduced into groups of naïve sentinels (S3 Fig). While there was evidence of transfer of the original PT21/28 strain 9000 (*stx2a*::ISEc8) isolate from the Trojan calves to the sentinels, only 1/10 super-shedding animal was established. By contrast after restoring Stx2a production in this PT21/28 strain (9000R) it was successfully transmitted to 10/10 sentinel animals of which 6/10 became super-shedders. On analysis the sentinel animals therefore excreted significantly higher levels of the Stx2a^+^ restored strain. A critical point is that when excretion levels for the two PT21/28 strains was compared for animals orally dosed with high levels of the bacteria (10^9^), then all animals became colonized at super-shedding levels and there was no significant difference in overall excretion level. As a consequence, we consider that the advantage conferred by *stx2a* is more evident and relevant during our transmission experiments which potentially reflect more natural, lower dose, exposure conditions with respect to the sentinel animals.

The oral challenge experiments also demonstrated that both PT21/28 strains 9000 and 9000R were excreted at significantly higher levels than the selected PT32 (strain 10671) isolate. It is therefore evident that Stx2a alone cannot account for the increased excretion phenotype of PT21/28 strains compared with PT32 strain 10671 when the infection dose is high. Alignment of the PacBio sequences for strains PT21/28 strain 9000 and PT32 strain 10671 examined in this study indicates that while a primary difference is the integrated ΦStx2a (S6 Fig), other differences in prophage regions, 2829 SNP differences and a total of 315 and 188 genes unique to strains 9000 and 10671 (S1 Table), respectively, were also detected. We have previously shown how protein regulators and sRNAs from the Stx2a encoding prophage can impact on other chromosomal loci, including type III secretion [42, 57]. Other ΦStx2a encoded genes and/or regulators therefore cannot be excluded from having a role in establishing a super-shedding phenotype. Future work will assess deletion of specific ΦStx2a and non-ΦStx2a regions *in vitro* before confirmatory experiments could be justified in cattle.

A definitive role for Stx in ruminants has been elusive with evidence for multiple phenotypes including adherence, immune modulation and killing of protozoa that predate on *E*. *coli* [19, 22, 23, 58]. In the present study we found no evidence to support a role for immune suppression [23] in the increased excretion of the Stx2a+ isolate by sentinel calves and in fact the strain with the reinstated *stx2a* generally exhibited higher responses to key antigens commensurate with higher excretion levels from these animals. Our research has demonstrated another potential role for Stx in the bovine host as supernatants containing Stx2a and Stx2c restricted the budding activity of bovine ileal organoid cultures. These cultures were set up to allow stem-like cells in the epithelial crypts to proliferate *in vitro* producing new epithelium which buds out from the seeded organoid [59]. Proliferation was determined by measuring an increase in the size of individual organoids and was restricted by cultures containing either Stx2a or Stx2c (or both) but there was no inhibition from induced culture supernatants in which no Stx toxin was present. This indicates that both Stx2a and Stx2c subtypes can prevent regeneration of the epithelium and provides evidence for an important phenotype that would have obvious benefits for bacteria that colonise by tight attachment to epithelial cells, as is the case for *E*. *coli* O157 at the terminal rectum of cattle [60].

**Fig 6:**
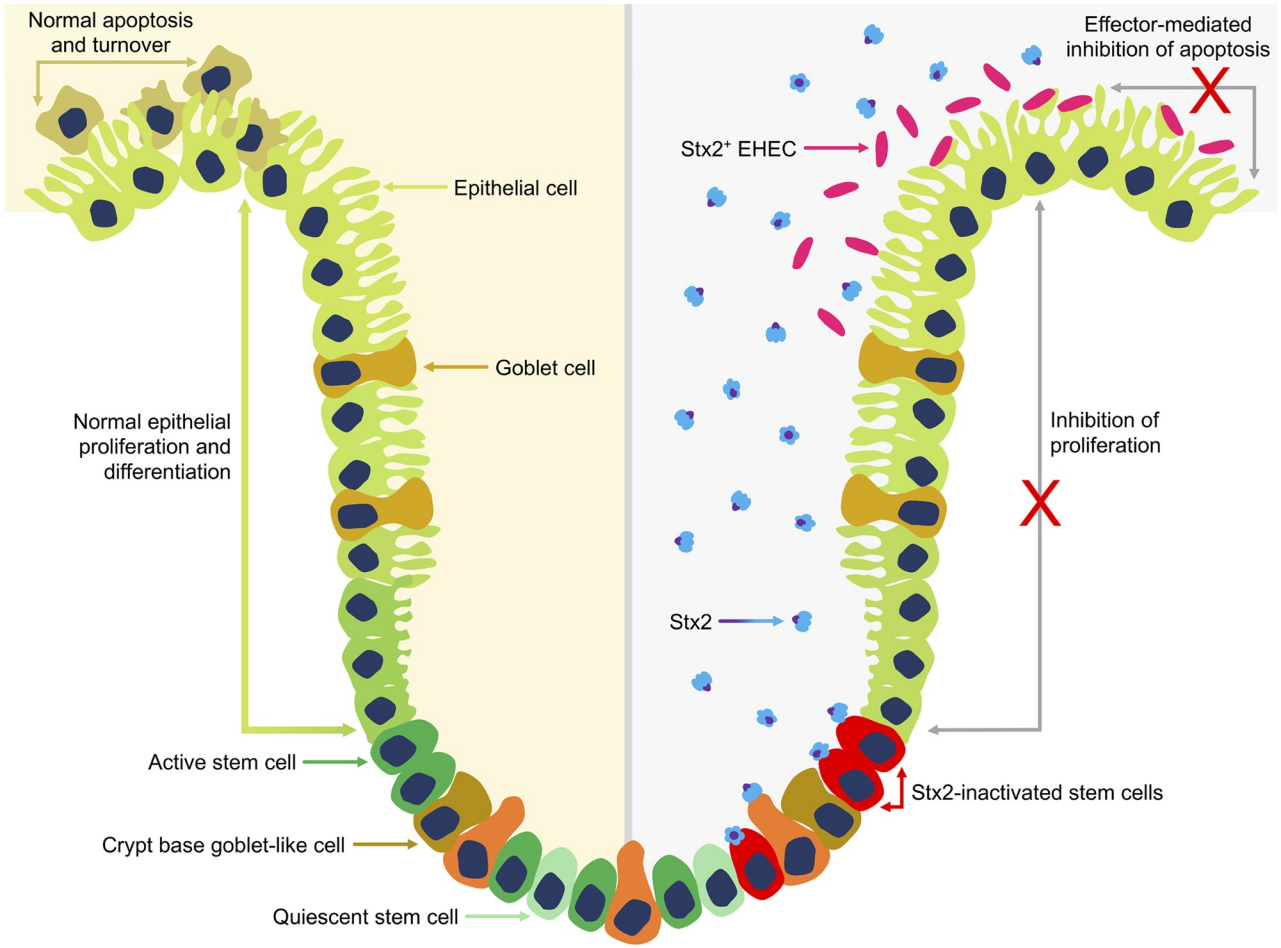
Stx2 mediated inhibition of bovine intestinal epithelial proliferation. Normal intestinal epithelial proliferation and differentiation (left) is driven by active stem cells located at the base of crypts. Stem cells continuously generate proliferating progeny that differentiate into the various cell lineages of mature intestinal villi and migrate up the crypt toward the villus tip. Continuous generation of new intestinal epithelial cells is balanced by apoptosis of older cells at the luminal surface resulting in the rapid turnover of intestinal epithelial cells. Expression and release of Stx2 into the intestinal crypt (right) by colonising Stx2+ EHEC O157 is proposed to inhibit normal stem cell driven proliferation. Inhibition may be direct by Stx2 binding to and inactivating Gb3+ stem cells or indirect inhibition of stem cells by interaction of Stx2 with as yet unidentified Gb3+ cells in the base of the crypt. *E*. *coli* O157 colonises at the luminal surface and further inhibits local epithelial turnover by type 3 secretion system-mediated delivery of specific effectors that can inhibit apoptosis.

Fig 6 illustrates a model for this Stx activity in which cell turnover is restricted in the intestinal epithelium by Stx2, presumably by activity on stem cells that drive epithelial expansion. This specific activity now needs to be determined but our previous research indicates that a subset of cells in bovine intestinal crypts are Gb3/CD77 positive (the receptor for Stx subtypes) and these may be stem-like cells [46, 47]. However, our previous work had indicated that purified Stx1 interaction with these receptors led to retrograde transport of the toxin out of the cell [47] and so it remains to be determined whether Stx2-subtypes have a different interaction or if Stx toxins are delivered in a different way, for example in outer membrane vesicles (OMVs) [61–63], they may avoid such retrograde trafficking. As proposed by others, it is also possible that Stx-subtypes can enhance colonisation by up-regulation of receptors for intimin such as nucleolin and integrin, although this would need a delivery mechanism, such as OMVs, into differentiated epithelial cells which are Gb3- in cattle [18, 19, 64].

The proposed inhibition of proliferation by Stx could work in conjunction with effector proteins that inhibit apoptosis as together they would help stabilise the colonised epithelium [65–67] (Fig 6). Of note the cycle-inhibiting factor (Cif) effector protein, produced by some specific sub-clusters of enteropathogenic *E*. *coli* (EPEC) and non-O157 EHEC, has been shown to cause cell cycle arrest and delays apoptosis when translocated into host cells [68, 69]. For *Shigella*, secreted effector proteins also act to stabilize the epithelium either by inhibiting cell proliferation (IpaB) [70] or cell removal (OspE) [71] to enable persistence of the colonising bacteria.

Predictive modelling previously concluded that Stx2a was likely to be a critical factor in the development of super-shedding and the occurrence of clinical human EHEC O157 isolates [28]. Furthermore super-shedding of EHEC O157 from cattle was predicted to significantly enhance the risk of infection in humans [28]. We have experimentally confirmed that Stx2a has a critical role in the development of a super-shedding phenotype and transmission of PT21/28 isolates. Although the exact mechanism by which Stx2a mediates a super-shedding phenotype remains unclear we propose that this is, in part, due to Stx2a being the dominant toxin produced by PT21/28 *Stx2a*^+^/*stx2c*^+^ strains in the GI tract of cattle and therefore has greater activity than Stx2c on the epithelium. Factors driving toxin production *in vivo* however are unknown and recent work has highlighted the complexity of Stx toxin production dynamics *in vivo* [72] including the possibility that phage induction does not require lysis for Stx release. Further work is therefore required to understand the expression and lysis dynamics of Stx2 phage and determine the relative levels of Stx2 subtypes produced during colonisation of hosts. It is also evident that super-shedding is multi-factorial, enabled by both Stx2a and the genetic background of the strain. Identifying PT21/28 specific genes or gene variants, in addition to stx2a, that are involved in super-shedding is essential to understanding this important phenotype.

Recent evidence indicates a subset of PT21/28 isolates associated with severe disease that have now lost the Stx2c prophage and consequently produce only the Stx2a toxin [10]. Our data demonstrates that such strains produce higher levels of Stx2a than strains encoding both Stx2a and Stx2c with increased activity for strain 9000RΔ*stx2c* on organoid budding. It remains a concern that as these bacteria evolve ways to increase the activity of Shiga toxins *in vivo* then such strains may represent more of a threat to human health. As a counter argument, detection of insertion sequence elements in *stx* genes is relatively common [73] therefore bacteria must encounter conditions where preventing expression of the toxin is a selective advantage. Inactivation of *stx2a* by three separate IS elements (IS1203v, IS629 and ISEc8) has now been reported [41, 73, 74] and selection against *stx*-encoding genes has been observed at farm-wide level [56]. Taken together, research in this area highlights the value of understanding Stx activity and drivers for its selection and maintenance in the animal reservoir as these should inform strategies that can reduce the threat to human health.

## Materials and methods

### Bacterial strains, culture conditions and inocula

Bacterial strains and primers used in this study are listed in Table 1 and S2 Table, respectively. Strain 9000R was constructed by removal of ISEc8 from *stx2a* in two steps by allelic exchange as described previously [75]. Primer pairs No stx2a/Ni stx2a and Co stx2a/Ci stx2a were used to generate PCR products of the 5’ and 3’ *stx2a* flanking regions, respectively. Products were cloned into pTOF25 and a Kan^R^ cassette was inserted between the *stx2a* flanking regions. This construct was used as an allelic exchange vector for deletion of *stx2a*. A wildtype *stx2a* gene with flanking regions was then generated by overlap extension PCR using primer pairs No stx2a/NiOE stx2a and Co stx2a/CiOE stx2a. The wildtype stx2a gene PCR product was cloned into pTOF25 and used as an allelic exchange vector to replace the Kan^R^ cassette with wildtype *stx2a* on the strain 9000 chromosome. Strain 9000 derivatives spontaneously cured of Φstx2a or Φstx2c lysogens were generated previously using an in-house selection method [41]. Deletion of ISEc8 and loss Φstx2a or Φstx2c were confirmed by PCR and sequencing. Nal^R^ derivative strains used in animal studies were naturally derived. All strains were cultured in lysogeny broth (LB) at 37 °C with shaking (200 r.p.m) unless otherwise stated. To prepare animal challenge inocula, Nal^R^ derivatives of strains 9000, 9000R or 10671 were resuscitated from freezer stocks on LB-agar plates and incubated at 37 °C overnight. Four 5 ml LB starter cultures were inoculated with single colonies (2 colonies per starter culture) and grown for 6 h (37 °C, 180 r.p.m.). After 6 h, starter cultures were pooled and used to inoculate (1/1000) 50 ml LB final inocula cultures. Final inocula cultures were grown for 18 h (37 °C, 200 r.p.m) before being used in animal studies.

### Ethics statement

All animal challenge experiments were performed at the Moredun Research Institute (MRI) under Home Office Licence 70/7914 granted by the UK Home Office under the Animal (Scientific Procedures) Act 1986. Ethical approval was obtained from the MRI Animal Experiments and Ethical Review Committee.

### Animal experiments

Transmission studies: Calves used were conventionally reared male Holstein-Friesian dairy cows with an average age of 12 ± 2 weeks at the time of challenge. Calves were fully weaned and fed hay and calf concentrate for 3-weeks prior to challenge, allowing for the establishment of ruminal flora. For each trial calves were randomly assigned to three rooms (C1 –C3) at the MRI High Security Unit (HSU). All calves were screened weekly and confirmed negative for EHEC O157:H7 by immunomagnetic separation (IMS) (anti-EHEC O157 Dynabeads; ThermoFisher) and qPCR (below) for four weeks prior to trial start. The experimental study design is shown in Supplementary S3 Fig. Calves housed in C1 were orally challenged on Day 0 by orogastric intubation with 500 ml PBS containing 10 ml of Nal^R^ final inocula cultures (Trial 1: Strain 10671, Trial 2: Strain 9000, Trial 3: Strain 9000R). At peak shedding (Day 5 post challenge) a high shedding calf (> 10^4^ cfu/g faeces) was moved into room C2 and C3 each housing naïve sentinel animals. Faecal shedding was monitored in all challenged and sentinel animals over a 25-day and 18-day period, respectively. 10 g faeces taken directly from the rectum was suspended in 90 ml PBS. Tenfold serial dilutions were made in PBS and 100 μl from three dilutions across a 1000-fold range was plated in triplicate on cefixime-tellurite sorbitol MacConkey (CT-SMAC) agar supplemented with Nalidixic acid (20 μg/ml). Re-suspended faeces were stored at 4 °C. Plates were incubated overnight at 37 °C and colonies enumerated at an appropriate dilution. Five to 10 colonies from each plate were confirmed O157 positive using *E*. *coli* O157 Latex Test kit (ThermoFisher). Where no colonies were observed, samples were enriched by adding 1 ml of re-suspended feces to 9 ml Tryptone Soya Broth (TSB; Oxoid). Samples were incubated at 37 °C overnight without shaking and plated onto CT-SMAC plates supplemented with Nalidixic acid (20 μg/ml). Bacterial growth was tested for the presence of O157 by latex agglutination. Faeces negative by direct plating but positive after enrichment were assigned an arbitrary value of 10 cfu/g.

For each of the three trials, a control group of age, sex and breed matched EHEC O157 negative calves (n = 5 per trial) was included to provide negative control material (serum, lymph node cells) for subsequent immunological assays. To monitor EHEC O157 shedding in these control calves, faecal samples were collected every second day for the duration of each trial. Samples were diluted 1:10 in PBS then plated out directly onto CT-SMAC plates and incubated overnight at 37 °C. No EHEC O157 colonies were detected in any of the control faecal samples tested.

### Quantitation of *E*. *coli* O157-specific antibody responses

Levels of *E*. *coli* O157-specific IgA, IgG_1_ and IgG_2_ were quantified in serum samples collected weekly from orally challenged or unchallenged control calves, or within antibody secreting cell (ASC) probes generated from rectal lymph node collected at post-mortem. These ASC probes represent spontaneous antibody production of antibodies from B cells within the rectal lymph nodes [52] and were generated as follows: rectal lymph nodes were collected and placed in transport medium (Hanks Balanced Salt Solution (HBSS) without calcium and magnesium, 2% heat inactivated foetal calf serum (Hi-FCS), 10 mg/ml gentamycin (Sigma-Aldrich), 200 IU/ml penicillin and 200 μg/ml streptomycin) prior to processing. Lymph nodes were washed twice in transport medium then cut into small (~0.5 cm^2^) pieces prior to being homogenized in a stomacher (Colwarth Stomacher 870, Seward Ltd, UK) for 30 s. The homogenized lymph node was then filtered through a 70 μm filter (Thermo-Fisher) before being under-laid with Ficoll-paque Plus (GE Healthcare) and centrifuged for 30 min at 800 × *g*. The mononuclear cell layer was washed twice with PBS, re-suspended in complete culture medium (RPMI 1640 (Gibco), 10% Hi-FCS, 200 mM L-glutamine, 50 μM β-mercaptoethanol, 200 IU/ml penicillin and 200 μg/ml streptomycin). Cells were then seeded into 24-well plates at a density of 5 x 10^6^ cells per well and incubated at 37°C and 5% CO_2_. After 5 d, supernatants containing spontaneously released antibody were collected and stored at -20°C prior to analysis.

Levels of IgA, IgG_1_ and IgG_2_ to four *E*. *coli* O157 antigens (H7, Tir, EspA and Intimin) were quantified by indirect ELISA as previously described [50] with results expressed as optical density at 492 nm (OD_492_). Optimal serum dilutions were determined following serial dilution of serum to ensure that the OD_492_ was on the linear part of the curve. Serum dilutions of 1:10 and 1:50 were used for IgA and IgG2 ELISAs respectively. For IgG1 ELISAs serum was diluted 1:100 for H7 and Intimin-specific ELISAs, 1:50 for EspA-specific ELISAs, and 1:250 for Tir-specific ELISAs. All ASC probes were analysed neat.

### Phage lysis curves and Stx toxin ELISA

3 ml LB was inoculated directly from glycerol stocks and grown overnight at 37 °C. 6 ml LB was inoculated 1/100 from overnight cultures and grown to an OD_600nm_ = 0.6–0.8 (t = 0). Cultures were split 1:1 and phage lysis was induced in one half by the addition of Mitomycin C (MMC, 2 μg/ml). Subsequent growth/lysis in induced and un-induced cultures was monitored spectrophotometrically at OD_600nm_. For Stx toxin ELISA assays cultures were grown as above and growth/lysis allowed to proceed for 24 h. After 24 h, 1 ml culture was taken and live cells and cell debris removed by centrifugation (13,000 r.p.m, room temperature (RT)). Stx toxin containing supernatants were further sterilized by syringe filtering (0.22 μm; Milipore). The level of Stx toxin in each sample was assayed using the RIDASCREEN Verotoxin ELISA kit (R-Biopharm) according to manufacturer guidelines.

### Culture of bovine ileal organoids

Bovine ileal organoids were derived previously from small intestinal crypts [48]. For toxin sensitivity assays ≈ 1,000 intestinal crypts in 100 μl advanced DMEM/F12 DMEM/F12 containing 1X B27 supplement minus vitamin A (ThermoFisher Scientifc), 25 μg/mL gentamicin and 100 U/ mL penicillin/streptomycin were added to 150 μl BD Growth Factor Reduced Matrigel Matrix (BD Biosciences, UK). 50 μl of Matrigel containing crypts were plated into the wells of a pre-warmed 24-well plate (NUNC, Thermo-Fisher) and incubated at 37 °C in a 5% CO_2_/air atmosphere for 10 min to allow the Matrigel to solidify. Once solidified 650 μl of pre-warmed IntestiCult Organoid Growth Medium (Mouse) (STEMCELL Technologies, UK) containing 50 μg/mL gentamicin and supplemented with 10 μM ROCK inhibitor (Y-27632, Cambridge Bioscience, UK), 500 nM TGF-β receptor kinase type 1 inhibitor (LY2157299, Cambridge Bioscience) and 10 μM p38 MAP kinase inhibitor (SB202190,Enzo Life Sciences, UK) was added. Stx toxin containing supernatants, derived as per ELISA assay, were added to organoids at a final dilution of 1/200 and organoids were maintained at 37 °C in a 5% CO_2_/air atmosphere replacing growth media every 2–3 days. 3D-organoids were imaged after 7-days using a Zeiss StereoLumar V12 Fluorescent Stereomicroscope (0.8x Objective Lens, 28x Zoom) and organoid size (in pixels) was determined using OrganSeg software with default settings [77]. For fluorescent cell imaging organoids were stained sequentially with Propidium Iodide (eBioscience) (dead cells) or Hoescht 33342 (all cells) at 7-days post treatment. Organoids were incubated first with 10 μl of propidium iodide added directly to the culture medium for 10 min, washed (2 x 650 μl of warm PBS) and then incubated with Hoescht 33342 diluted 1/1000 in 650 μl PBS for 10 min before fixation with freshly-prepared 1% paraformaldehyde (PFA). Stained organoids were imaged using a Zeiss Axiovert 25 inverted fluorescent microscope. Organoids were routinely passaged after 7-days by removing growth media followed by resuspension of Matrigel in 1 ml ice-cold advanced DMEM/F12 containing 1X B27 supplement minus vitamin A (ThermoFisher Scientifc), 25 μg/mL gentamicin and 100 U/mL penicillin/streptomycin by pipetting. Resulting suspensions were transfer to a Pyrex FACS tube (Corning, Wycombe, UK) and organoids were allowed to settle. The supernatant was removed, organoids were re-suspended in 1 ml advanced DMEM/F12 and then mechanically disrupted by pipetting using a 200 μl pipette tip. The number of crypts was counted and organoid cultures were established as above.

### Multiplex qPCR screening and RT-qPCR

All calves were pre-screened for the presence of EHEC O157:H7 by qPCR on a weekly basis for four weeks prior to trial start. 1 g of faeces, taken directly from the rectum, was suspended in 10 ml EC broth (Oxoid) supplemented with Novobiocin (15 μg/ml) and incubated statically at 37 °C for 6 h. After 6 h, 100 μL of enriched culture was suspended in 900 μl PBS, cells harvested by centrifugation (13,000 r.p.m, 5 min, RT) and total DNA isolated using InstaGene Matrix (Bio-Rad) according to manufacturer guidelines. Isolated DNA was screened for the presence of the EHEC O157:H7 *rfb* gene and Stx toxin variants *stx1* and *stx2* by multiplex qPCR using primer probe pairs (IDT DNA) specific for each gene (S2 Table). All reactions were carried out using a QuantiTect Probe PCR kit (Qiagen) according to manufacturer guidelines under the following conditions: 95 °C for 15 min (1 cycle), 95 °C, 15 sec; 60 °C, 1 min (45 cycles). Expression of *N*, *cro* and *stx* from Φstx2a and Φstx2c was monitored by RT-qPCR during lysis. Cultures were grown as per lysis curves and 0.2 OD_600nm_ culture units were harvested at t = 0, 30, 60, 90 and 120 min by centrifugation. Total RNA was extracted from cell pellets using a RNeasy Mini kit (Qiagen) according to manufacturer guidelines. Extracted RNA was quantified and 2 μg of each samples was DNase treated using TURBO DNA-*free* kit. 200 ng of DNase treated RNA was then converted to cDNA using iScript Reverse Transcription Supermix (Bio-Rad) according to manufacturer guidelines. All qPCR reactions were carried out using iQ Syber Green supermix (Bio-Rad) and gene specific primers (IDT-DNA) under the following conditions: 95 °C for 15 s (1 cycle), 95 °C for 15 s; 60 °C for 1 min (40 cycles). Gene expression was quantified relative to a standard curve generated from 9000R genomic DNA.

### Genomic sequence comparison

Whole genome chromosomal sequences of strains 9000 and 10671 were obtained from NCBI (accession numbers: CP018252.1, and CP018250.1) as full Genbank files, and complete nucleotide sequence FASTA files. FASTA files were used to make a ProgressiveMauve alignment [78], and the SNPs were extracted using the export SNPs option. FASTA files were also used in conjunction with the prophage sequences of strain 9000 [41], to produce a BLAST Ring Image Generator (BRIG) diagram [79] displaying the content of strain 9000 absent, or different in strain 10671 (blast+ version 2.2.31+) (S3 Fig). Genbank files were converted to GFF3 files using the BioPerl [80] script genbank2gff3 and input into Roary [81] to obtain the core and shell genes for each strain. The gene presence and absence file was then amended with gene loci to show *bona fide* gene content differences between strains 9000 and 10671 (S1 Table).

### Statistical analysis

Analysis of daily shedding measured in cfu/g from challenged and sentinel animals was conducted by fitting separate Poisson generalised linear mixed models (GLMM) by the maximum likelihood method, using logarithmic link function and Laplace approximations to calculate log-likelihoods. The models included the logarithm of the corresponding dilution factor as an offset variable. The fixed effects part of the models consisted of bacterial strain, post-challenge day (centred at the mean day, 11.69 and 9.3 for challenged and sentinel animals respectively) and a term accounting for the interaction both. The relationship between repeated measurements from the same animal was accounted for by including animal as a random effect. Random effects of pen and of the interaction between animal and day were included in the model along with a random effect at observation level to account for data over-dispersion. Total shedding over the course of the experiment was estimated for each animal by the area under the curve (AUC) using the composite trapezoid rule. Differences in mean AUCs per strain were statistically tested by fitting negative binomial generalised linear models (GLM) by iteratively reweighted least squares (IWLS) using a logarithm link function. This way we considered an extra parameter to model over-dispersion in AUC. For sentinels, all animals were negative to strain 10671 and, hence, only those positive to strains 9000 and 9000R were considered in the modelling. For challenged animals, pairwise tests of differences in mean between strains were conducted based on the predicted marginal means from the GLMM and GLM estimates. The corresponding *p*-values were adjusted for multiplicity using the false discovery rate (FDR) approach [82]. Note that only data from day 1 were considered for fitting the models as the values were all zero at day 0.

PCR data were log transformed and differences in mean gene expression between genes encoded by ΦStx2a and ΦStx2c analogues were statistically assessed at each time-point using *t*-tests. The corresponding *p*-values were corrected for multiplicity using the FDR approach [82]. Stx2 production data was analysed by ordinary one-way ANOVA with multiple comparisons testing. Differences in mean organoid size were statistically assessed by ordinary one-way ANOVA multiple comparisons tests in which mean organoid size for each supernatant treatment was compared with strain 9000 or untreated controls. The corresponding *p*-values were corrected for multiplicity using the FDR approach [82].

Antibody data was analysed as follows: non-parametric Mann-Whitney *U* tests were used to compare antibody levels within ASC probes. Generalised additive mixed models with identity link function and Gaussian errors were fitted by REML to investigate the effects of challenge strain on serum antibody responses over time. The antibody responses were (log + 1) transformed to normalise the data. The models included challenge strain as a fixed effect and spline-based smooth terms (one per strain) to account for potential non-linear relationships of the response with time, and animal added as a random effect. Heterogeneous variances by group were allowed.

Statistical modelling of bacterial shedding, antibody data over time and transmission data was conducted on the R system for statistical computing version 3.2 [83]. PCR data, Stx2 production data and ASC antibody data were analysed using GraphPad Prism version 6.05 for Windows (GraphPad Software, La Jolla California USA, www.graphpad.com). In all cases statistical test significance was assessed at the 5% significance level.

## Supporting information

S1 Fig Genomic context of *stx2A* gene in strains 9000 and 9000R. Adapted alignment of strains 9000 and 9000R sequences shows the respective presence and absence of ISEc8 in the *stx2a* A subunit. Genomes (black lines), named genes (coloured blocks) and regions of homology (indigo lines) are shown.
(TIF)

S2 Fig Viability staining of bovine ileal organoids following treatment with Stx2. Untreated organoids and organoids treated with Stx2-containing supernatants from *E*. *coli* O157 strains 9000 (*stx2c*+ *stx2a*::ISEc8) and 9000R (*stx2c*+ *stx2a*) were fluorescently stained for all cells (blue) and dead cells (green) using Hoescht 33342 and Propidium Iodide, respectively. Representative paired phase contrast and fluorescence images of organoids are shown in triplicate for each treatment.
(TIF)

S3 Fig Experimental design for *E*. *coli* O157 transmission studies. (A) Uninfected naïve calves (n = 5 per room) were housed in rooms C1, C2 and C3. (B) On day 0 all calves in room C1 were experimentally infected with ~10^9^ CFU of *E*. *coli* O157 by orogastric intubation. (C) At five days post-challenge a calf shedding >10^4^ cfu/g faeces was moved into rooms C2 and C3, respectively. Faecal bacterial shedding (cfu/g) and environmental levels were monitored daily for a further 18-day period.
(PDF)

S4 Fig Shedding curves for animals colonized with *E*. *coli* O157 strains 10671, 9000 and 9000R. Shedding (cfu/g faeces) of PT32 strain 10671 and PT21/28 strains 9000 and 9000R was monitored from experimentally infected animals (Room C1) and sentinel animals (Rooms C2 and C3). Environmental bacterial levels within each room (blue) and shedding from colonised Trojan animals (red) in rooms C2 and C3 are also shown. The average cfu/g faeces (for individual calves) or cfu/g environmental material from three replicate plate counts are plotted.
(TIF)

S5 Fig Weekly serum antibody responses to strains 9000, 9000R and 10671. Serum levels of (A) H7-specific; (B) Tir-specific; (C) EspA-specific and (D) Intimin-specific serum antibody levels in *E*. *coli* O157 challenged and unchallenged control calves. Levels of antigen-specific IgA, IgG_1_ and IgG_2_ in weekly serum samples collected from calves orally challenged with ~10^9^ CFU *E*. *coli* O157 strains 9000, 9000R or 10671, or from unchallenged control calves were determined by indirect ELISA. Data represents the mean value ± SEM.
(PDF)

S6 Fig BRIG plot comparing *E*. *coli* O157 strains 9000 and 10671. The genome of PT32 strain 10671 (red) was compared against reference PT21/28 strain 9000 (blue) for gene presence/absence. Annotated prophage (grey) and their loci, including Stx2aΦ centred at 3,200 kbp, are shown for strain 9000.
(TIF)

S1 Table List of genes unique to *E*. *coli* O157 strains 9000 and 10671. (XLSX)

S2 Table Details of PCR primers used in this study. (DOCX)

## References

[1] Obrig TG, Karpman D. Shiga toxin pathogenesis: kidney complications and renal failure. Current topics in microbiology and immunology. 2012;357:105–36. 10.1007/82_2011_172 21983749 PMC3779650

[2] Garcia A, Fox JG, Besser TE. Zoonotic enterohemorrhagic *Escherichia coli*: A One Health perspective. ILAR journal / National Research Council, Institute of Laboratory Animal Resources. 2010;51(3):221–32. 10.1093/ilar.51.3.221 . 21131723

[3] Majowicz SE, Scallan E, Jones-Bitton A, Sargeant JM, Stapleton J, Angulo FJ, et al Global incidence of human Shiga toxin-producing *Escherichia coli* infections and deaths: a systematic review and knowledge synthesis. Foodborne pathogens and disease. 2014;11(6):447–55. 10.1089/fpd.2013.1704 24750096 PMC4607253

[4] Rangel JM, Sparling PH, Crowe C, Griffin PM, Swerdlow DL. Epidemiology of *Escherichia coli* O157:H7 outbreaks, United States, 1982–2002. Emerging infectious diseases. 2005;11(4):603–9. 10.3201/eid1104.040739 15829201 PMC3320345

[5] Melton-Celsa A, Mohawk K, Teel L, O’Brien A. Pathogenesis of Shiga-toxin producing *Escherichia coli*. Current topics in microbiology and immunology. 2012;357:67–103. 10.1007/82_2011_176 . 21915773

[6] Melton-Celsa AR. Shiga Toxin (Stx) Classification, Structure, and Function. Microbiology spectrum. 2014;2(2). 10.1128/microbiolspec.EHEC-0024-2013 25530917 PMC4270005

[7] Scheutz F, Teel LD, Beutin L, Pierard D, Buvens G, Karch H, et al Multicenter evaluation of a sequence-based protocol for subtyping Shiga toxins and standardizing Stx nomenclature. Journal of clinical microbiology. 2012;50(9):2951–63. 10.1128/JCM.00860-12 22760050 PMC3421821

[8] Brandal LT, Wester AL, Lange H, Lobersli I, Lindstedt BA, Vold L, et al Shiga toxin-producing *Escherichia coli* infections in Norway, 1992–2012: characterization of isolates and identification of risk factors for haemolytic uremic syndrome. BMC infectious diseases. 2015;15:324 10.1186/s12879-015-1017-6 26259588 PMC4531490

[9] Buvens G, De Gheldre Y, Dediste A, de Moreau AI, Mascart G, Simon A, et al Incidence and virulence determinants of verocytotoxin-producing *Escherichia coli* infections in the Brussels-Capital Region, Belgium, in 2008–2010. Journal of clinical microbiology. 2012;50(4):1336–45. 10.1128/JCM.05317-11 22238434 PMC3318570

[10] Dallman TJ, Ashton PM, Byrne L, Perry NT, Petrovska L, Ellis R, et al Applying phylogenomics to understand the emergence of Shiga-toxin-producing *Escherichia coli* O157: H7 strains causing severe human disease in the UK. Microbial Genomics. 2015;1(3). 10.1099/mgen.0.000029 PMC5320567 28348814

[11] Fuller CA, Pellino CA, Flagler MJ, Strasser JE, Weiss AA. Shiga toxin subtypes display dramatic differences in potency. Infection and immunity. 2011;79(3):1329–37. 10.1128/IAI.01182-10 21199911 PMC3067513

[12] Louise CB, Obrig TG. Specific interaction of *Escherichia coli* O157:H7-derived Shiga-like toxin II with human renal endothelial cells. The Journal of infectious diseases. 1995;172(5):1397–401. 10.1093/infdis/172.5.1397 . 7594687

[13] Russo LM, Melton-Celsa AR, O’Brien AD. Shiga Toxin (Stx) Type 1a Reduces the Oral Toxicity of Stx Type 2a. The Journal of infectious diseases. 2016;213(8):1271–9. 10.1093/infdis/jiv557 26743841 PMC4799663

[14] Kawano K, Okada M, Haga T, Maeda K, Goto Y. Relationship between pathogenicity for humans and stx genotype in Shiga toxin-producing *Escherichia coli* serotype O157. European journal of clinical microbiology & infectious diseases: official publication of the European Society of Clinical Microbiology. 2008;27(3):227–32. 10.1007/s10096-007-0420-3 . 18071766

[15] Fogg PC, Saunders JR, McCarthy AJ, Allison HE. Cumulative effect of prophage burden on Shiga toxin production in Escherichia coli. Microbiology. 2012;158(Pt 2):488–97. 10.1099/mic.0.054981-0 . 22096150

[16] de Sablet T, Bertin Y, Vareille M, Girardeau JP, Garrivier A, Gobert AP, et al Differential expression of stx2 variants in Shiga toxin-producing *Escherichia coli* belonging to seropathotypes A and C. Microbiology. 2008;154(Pt 1):176–86. 10.1099/mic.0.2007/009704-0 . 18174136

[17] Ogura Y, Mondal SI, Islam MR, Mako T, Arisawa K, Katsura K, et al The Shiga toxin 2 production level in enterohemorrhagic *Escherichia coli* O157:H7 is correlated with the subtypes of toxin-encoding phage. Scientific reports. 2015;5:16663 10.1038/srep16663 26567959 PMC4645166

[18] Robinson CM, Sinclair JF, Smith MJ, O’Brien AD. Shiga toxin of enterohemorrhagic *Escherichia coli* type O157:H7 promotes intestinal colonization. Proceedings of the National Academy of Sciences of the United States of America. 2006;103(25):9667–72. 10.1073/pnas.0602359103 16766659 PMC1475797

[19] Liu B, Yin X, Feng Y, Chambers JR, Guo A, Gong J, et al Verotoxin 2 enhances adherence of enterohemorrhagic *Escherichia coli* O157:H7 to intestinal epithelial cells and expression of {beta}1-integrin by IPEC-J2 cells. Appl Environ Microbiol. 2010;76(13):4461–8. 10.1128/AEM.00182-10 20453145 PMC2897442

[20] Gobert AP, Coste A, Guzman CA, Vareille M, Hindre T, de Sablet T, et al Modulation of chemokine gene expression by Shiga-toxin producing *Escherichia coli* belonging to various origins and serotypes. Microbes and infection. 2008;10(2):159–65. 10.1016/j.micinf.2007.10.018 . 18248761

[21] Gobert AP, Vareille M, Glasser AL, Hindre T, de Sablet T, Martin C. Shiga toxin produced by enterohemorrhagic *Escherichia coli* inhibits PI3K/NF-kappaB signaling pathway in globotriaosylceramide-3-negative human intestinal epithelial cells. Journal of immunology. 2007;178(12):8168–74. 10.4049/jimmunol.178.12.8168 . 17548655

[22] Hoffman MA, Menge C, Casey TA, Laegreid W, Bosworth BT, Dean-Nystrom EA. Bovine immune response to shiga-toxigenic *Escherichia coli* O157:H7. Clinical and vaccine immunology: CVI. 2006;13(12):1322–7. 10.1128/CVI.00205-06 17050743 PMC1694447

[23] Menge C, Wieler LH, Schlapp T, Baljer G. Shiga toxin 1 from *Escherichia coli* blocks activation and proliferation of bovine lymphocyte subpopulations in vitro. Infection and immunity. 1999;67(5):2209–17. 10225876 10.1128/iai.67.5.2209-2217.1999 PMC115959

[24] Stamm I, Mohr M, Bridger PS, Schropfer E, Konig M, Stoffregen WC, et al Epithelial and mesenchymal cells in the bovine colonic mucosa differ in their responsiveness to *Escherichia coli* Shiga toxin 1. Infection and immunity. 2008;76(11):5381–91. 10.1128/IAI.00553-08 18765725 PMC2573328

[25] Steinberg KM, Levin BR. Grazing protozoa and the evolution of the *Escherichia coli* O157:H7 Shiga toxin-encoding prophage. Proceedings Biological sciences. 2007;274(1621):1921–9. 10.1098/rspb.2007.0245 17535798 PMC2211389

[26] Schmidt CE, Shringi S, Besser TE. Protozoan Predation of *Escherichia coli* O157:H7 Is Unaffected by the Carriage of Shiga Toxin-Encoding Bacteriophages. PloS one. 2016;11(1):e0147270 10.1371/journal.pone.0147270 26824472 PMC4732659

[27] Chase-Topping ME, McKendrick IJ, Pearce MC, MacDonald P, Matthews L, Halliday J, et al Risk factors for the presence of high-level shedders of *Escherichia coli* O157 on Scottish farms. Journal of clinical microbiology. 2007;45(5):1594–603. 10.1128/JCM.01690-06 17360845 PMC1865900

[28] Matthews L, Reeve R, Gally DL, Low JC, Woolhouse ME, McAteer SP, et al Predicting the public health benefit of vaccinating cattle against *Escherichia coli* O157. Proceedings of the National Academy of Sciences of the United States of America. 2013;110(40):16265–70. 10.1073/pnas.1304978110 24043803 PMC3791763

[29] Munns KD, Selinger LB, Stanford K, Guan L, Callaway TR, McAllister TA. Perspectives on super-shedding of *Escherichia coli* O157:H7 by cattle. Foodborne pathogens and disease. 2015;12(2):89–103. 10.1089/fpd.2014.1829 . 25514549

[30] Munns KD, Zaheer R, Xu Y, Stanford K, Laing CR, Gannon VP, et al Comparative Genomic Analysis of *Escherichia coli* O157:H7 Isolated from Super-Shedder and Low-Shedder Cattle. PloS one. 2016;11(3):e0151673 10.1371/journal.pone.0151673 27018858 PMC4809568

[31] Spencer SE, Besser TE, Cobbold RN, French NP. ‘Super’ or just ‘above average’? Supershedders and the transmission of *Escherichia coli* O157:H7 among feedlot cattle. Journal of the Royal Society, Interface / the Royal Society. 2015;12(110):0446 10.1098/rsif.2015.0446 26269231 PMC4614454

[32] Chase-Topping M, Gally D, Low C, Matthews L, Woolhouse M. Super-shedding and the link between human infection and livestock carriage of *Escherichia coli* O157. Nature reviews Microbiology. 2008;6(12):904–12. 10.1038/nrmicro2029 . 19008890 PMC5844465

[33] Omisakin F, MacRae M, Ogden ID, Strachan NJ. Concentration and prevalence of *Escherichia coli* O157 in cattle feces at slaughter. Applied and environmental microbiology. 2003;69(5):2444–7. 10.1128/AEM.69.5.2444-2447.2003 12732509 PMC154535

[34] Low JC, McKendrick IJ, McKechnie C, Fenlon D, Naylor SW, Currie C, et al Rectal carriage of enterohemorrhagic *Escherichia coli* O157 in slaughtered cattle. Applied and environmental microbiology. 2005;71(1):93–7. 10.1128/AEM.71.1.93-97.2005 15640175 PMC544206

[35] Stephens TP, McAllister TA, Stanford K. Perineal swabs reveal effect of super shedders on the transmission of *Escherichia coli* O157:H7 in commercial feedlots. Journal of animal science. 2009;87(12):4151–60. 10.2527/jas.2009-1967 . 19684276

[36] Matthews L, Low JC, Gally DL, Pearce MC, Mellor DJ, Heesterbeek JA, et al Heterogeneous shedding of *Escherichia coli* O157 in cattle and its implications for control. Proceedings of the National Academy of Sciences of the United States of America. 2006;103(3):547–52. 10.1073/pnas.0503776103 16407143 PMC1325964

[37] Cobbold RN, Hancock DD, Rice DH, Berg J, Stilborn R, Hovde CJ, et al Rectoanal junction colonization of feedlot cattle by *Escherichia coli* O157:H7 and its association with supershedders and excretion dynamics. Applied and environmental microbiology. 2007;73(5):1563–8. 10.1128/AEM.01742-06 17220263 PMC1828767

[38] Matthews L, McKendrick IJ, Ternent H, Gunn GJ, Synge B, Woolhouse ME. Super-shedding cattle and the transmission dynamics of *Escherichia coli* O157. Epidemiology and infection. 2006;134(1):131–42. 10.1017/S0950268805004590 16409660 PMC2870353

[39] Arthur TM, Ahmed R, Chase-Topping M, Kalchayanand N, Schmidt JW, Bono JL. Characterization of *Escherichia coli* O157:H7 strains isolated from supershedding cattle. Appl Environ Microbiol. 2013;79(14):4294–303. 10.1128/AEM.00846-13 23645203 PMC3697492

[40] Pearce MC, Chase-Topping ME, McKendrick IJ, Mellor DJ, Locking ME, Allison L, et al Temporal and spatial patterns of bovine *Escherichia coli* O157 prevalence and comparison of temporal changes in the patterns of phage types associated with bovine shedding and human *E*. *coli* O157 cases in Scotland between 1998–2000 and 2002–2004. BMC microbiology. 2009;9:276 10.1186/1471-2180-9-276 20040112 PMC2808314

[41] Shaaban S. C, L A., McAteer SP., Jenkins C., Dallman TJ., Bono JL., and Gally DL. Evolution of a zoonotic pathogen: investigating prophage diversity in enterohaemorrhagic *Escherichia coli* O157 by long-read sequencing. Microbial Genomics. 2016 10.1099/mgen.0.000096 28348836 PMC5359411

[42] Xu X, McAteer SP, Tree JJ, Shaw DJ, Wolfson EB, Beatson SA, et al Lysogeny with Shiga toxin 2-encoding bacteriophages represses type III secretion in enterohemorrhagic *Escherichia coli*. PLoS pathogens. 2012;8(5):e1002672 10.1371/journal.ppat.1002672 22615557 PMC3355084

[43] Lupolova N, Dallman T, Bono J, Gally D. Support Vector Machine applied to predict the zoonotic potential of *E*. *coli* O157 cattle isolates. Proceedings of the National Academy of Sciences. 2016 10.1073/pnas.1606567113 27647883 PMC5056084

[44] Gallegos KM, Conrady DG, Karve SS, Gunasekera TS, Herr AB, Weiss AA. Shiga toxin binding to glycolipids and glycans. PloS one. 2012;7(2):e30368 10.1371/journal.pone.0030368 22348006 PMC3278406

[45] Karve SS, Weiss AA. Glycolipid binding preferences of Shiga toxin variants. PloS one. 2014;9(7):e101173 10.1371/journal.pone.0101173 24983355 PMC4077739

[46] Hoey DE, Currie C, Else RW, Nutikka A, Lingwood CA, Gally DL, et al Expression of receptors for verotoxin 1 from *Escherichia coli* O157 on bovine intestinal epithelium. J Med Microbiol. 2002;51(2):143–9. 10.1099/0022-1317-51-2-143 . 11865842

[47] Hoey DE, Sharp L, Currie C, Lingwood CA, Gally DL, Smith DG. Verotoxin 1 binding to intestinal crypt epithelial cells results in localization to lysosomes and abrogation of toxicity. Cellular microbiology. 2003;5(2):85–97. . 12580945 10.1046/j.1462-5822.2003.00254.x

[48] Hamilton CA, Young R, Jayaraman S, Sehgal A, Paxton E, Thomson S, et al Development of in vitro enteroids derived from bovine small intestinal crypts. Vet Res. 2018;49(1):54 10.1186/s13567-018-0547-5 29970174 PMC6029049

[49] McNeilly TN, Naylor SW, Mahajan A, Mitchell MC, McAteer S, Deane D, et al *Escherichia coli* O157:H7 colonization in cattle following systemic and mucosal immunization with purified H7 flagellin. Infection and immunity. 2008;76(6):2594–602. 10.1128/IAI.01452-07 18362130 PMC2423056

[50] McNeilly TN, Mitchell MC, Rosser T, McAteer S, Low JC, Smith DG, et al Immunization of cattle with a combination of purified intimin-531, EspA and Tir significantly reduces shedding of *Escherichia coli* O157:H7 following oral challenge. Vaccine. 2010;28(5):1422–8. 10.1016/j.vaccine.2009.10.076 . 19903545

[51] Naylor SW, Low JC, Besser TE, Mahajan A, Gunn GJ, Pearce MC, et al Lymphoid follicle-dense mucosa at the terminal rectum is the principal site of colonization of enterohemorrhagic *Escherichia coli* O157:H7 in the bovine host. Infection and immunity. 2003;71(3):1505–12. 10.1128/IAI.71.3.1505-1512.2003 12595469 PMC148874

[52] Meeusen EN, Brandon MR. Antibody secreting cells as specific probes for antigen identification. J Immunol Methods. 1994;172(1):71–6. 10.1016/0022-1759(94)90379-4 . 8207267

[53] Soderlund R, Jernberg C, Ivarsson S, Hedenstrom I, Eriksson E, Bongcam-Rudloff E, et al Molecular typing of *Escherichia coli* O157:H7 isolates from Swedish cattle and human cases: population dynamics and virulence. Journal of clinical microbiology. 2014;52(11):3906–12. 10.1128/JCM.01877-14 25143581 PMC4313222

[54] Pianciola L, D’Astek BA, Mazzeo M, Chinen I, Masana M, Rivas M. Genetic features of human and bovine *Escherichia coli* O157:H7 strains isolated in Argentina. International journal of medical microbiology: IJMM. 2016;306(2):123–30. 10.1016/j.ijmm.2016.02.005 . 26935026

[55] Ashton PM, Perry N, Ellis R, Petrovska L, Wain J, Grant KA, et al Insight into Shiga toxin genes encoded by *Escherichia coli* O157 from whole genome sequencing. PeerJ. 2015;3:e739 10.7717/peerj.739 25737808 PMC4338798

[56] Park D, Stanton E, Ciezki K, Parrell D, Bozile M, Pike D, et al Evolution of the Stx2-encoding prophage in persistent bovine *Escherichia coli* O157:H7 strains. Applied and environmental microbiology. 2013;79(5):1563–72. 10.1128/AEM.03158-12 23275514 PMC3591979

[57] Tree JJ, Granneman S, McAteer SP, Tollervey D, Gally DL. Identification of bacteriophage-encoded anti-sRNAs in pathogenic *Escherichia coli*. Molecular cell. 2014;55(2):199–213. 10.1016/j.molcel.2014.05.006 24910100 PMC4104026

[58] Stanford K, Bach SJ, Stephens TP, McAllister TA. Effect of rumen protozoa on *Escherichia coli* O157:H7 in the rumen and feces of specifically faunated sheep. Journal of food protection. 2010;73(12):2197–202. 10.4315/0362-028x-73.12.2197 . 21219736

[59] Sehgal A, Donaldson DS, Pridans C, Sauter KA, Hume DA, Mabbott NA. The role of CSF1R-dependent macrophages in control of the intestinal stem-cell niche. Nature communications. 2018;9(1):1272 10.1038/s41467-018-03638-6 29593242 PMC5871851

[60] Naylor SW, Roe AJ, Nart P, Spears K, Smith DG, Low JC, et al *Escherichia coli* O157: H7 forms attaching and effacing lesions at the terminal rectum of cattle and colonization requires the LEE4 operon. Microbiology. 2005;151(Pt 8):2773–81. 10.1099/mic.0.28060-0 . 16079353

[61] Kolling GL, Matthews KR. Export of virulence genes and Shiga toxin by membrane vesicles of *Escherichia coli* O157:H7. Applied and environmental microbiology. 1999;65(5):1843–8. 10223967 10.1128/aem.65.5.1843-1848.1999 PMC91264

[62] Yokoyama K, Horii T, Yamashino T, Hashikawa S, Barua S, Hasegawa T, et al Production of shiga toxin by *Escherichia coli* measured with reference to the membrane vesicle-associated toxins. FEMS microbiology letters. 2000;192(1):139–44. 10.1111/j.1574-6968.2000.tb09372.x . 11040442

[63] Bielaszewska M, Ruter C, Bauwens A, Greune L, Jarosch KA, Steil D, et al Host cell interactions of outer membrane vesicle-associated virulence factors of enterohemorrhagic *Escherichia coli* O157: Intracellular delivery, trafficking and mechanisms of cell injury. PLoS pathogens. 2017;13(2):e1006159 10.1371/journal.ppat.1006159 28158302 PMC5310930

[64] Pruimboom-Brees IM, Morgan TW, Ackermann MR, Nystrom ED, Samuel JE, Cornick NA, et al Cattle lack vascular receptors for *Escherichia coli* O157:H7 Shiga toxins. Proceedings of the National Academy of Sciences of the United States of America. 2000;97(19):10325–9. 10.1073/pnas.190329997 10973498 PMC27023

[65] Blasche S, Mortl M, Steuber H, Siszler G, Nisa S, Schwarz F, et al The *E*. *coli* effector protein NleF is a caspase inhibitor. PloS one. 2013;8(3):e58937 10.1371/journal.pone.0058937 23516580 PMC3597564

[66] Scott NE, Giogha C, Pollock GL, Kennedy CL, Webb AI, Williamson NA, et al The bacterial arginine glycosyltransferase effector NleB preferentially modifies Fas-associated death domain protein (FADD). J Biol Chem. 2017;292(42):17337–50. 10.1074/jbc.M117.805036 28860194 PMC5655511

[67] Wong AR, Pearson JS, Bright MD, Munera D, Robinson KS, Lee SF, et al Enteropathogenic and enterohaemorrhagic *Escherichia coli*: even more subversive elements. Molecular microbiology. 2011;80(6):1420–38. 10.1111/j.1365-2958.2011.07661.x . 21488979

[68] Loukiadis E, Nobe R, Herold S, Tramuta C, Ogura Y, Ooka T, et al Distribution, functional expression, and genetic organization of Cif, a phage-encoded type III-secreted effector from enteropathogenic and enterohemorrhagic *Escherichia coli*. Journal of bacteriology. 2008;190(1):275–85. 10.1128/JB.00844-07 17873042 PMC2223761

[69] Samba-Louaka A, Nougayrede JP, Watrin C, Oswald E, Taieb F. The enteropathogenic *Escherichia coli* effector Cif induces delayed apoptosis in epithelial cells. Infection and immunity. 2009;77(12):5471–7. 10.1128/IAI.00860-09 19786559 PMC2786488

[70] Iwai H, Kim M, Yoshikawa Y, Ashida H, Ogawa M, Fujita Y, et al A bacterial effector targets Mad2L2, an APC inhibitor, to modulate host cell cycling. Cell. 2007;130(4):611–23. 10.1016/j.cell.2007.06.043 . 17719540

[71] Kim M, Ogawa M, Fujita Y, Yoshikawa Y, Nagai T, Koyama T, et al Bacteria hijack integrin-linked kinase to stabilize focal adhesions and block cell detachment. Nature. 2009;459(7246):578–82. 10.1038/nature07952 . 19489119

[72] Balasubramanian S, Osburne MS, BrinJones H, Tai AK, Leong JM. Prophage induction, but not production of phage particles, is required for lethal disease in a microbiome-replete murine model of enterohemorrhagic *E*. *coli* infection. PLoS pathogens. 2019;15(1):e1007494 10.1371/journal.ppat.1007494 . 30629725 PMC6328086

[73] Loftsdottir H, Soderlund R, Jinnerot T, Eriksson E, Bongcam-Rudloff E, Aspan A. Dynamics of insertion sequence element IS629 inactivation of verotoxin 2 genes in *Escherichia coli* O157:H7. FEMS microbiology letters. 2017 10.1093/femsle/fnx074 . 28402463

[74] Kusumoto M, Nishiya Y, Kawamura Y. Reactivation of insertionally inactivated Shiga toxin 2 genes of *Escherichia coli* O157:H7 caused by nonreplicative transposition of the insertion sequence. Applied and environmental microbiology. 2000;66(3):1133–8. 10.1128/aem.66.3.1133-1138.2000 10698782 PMC91953

[75] Merlin C, McAteer S, Masters M. Tools for characterization of *Escherichia coli* genes of unknown function. Journal of bacteriology. 2002;184(16):4573–81. 10.1128/JB.184.16.4573-4581.2002 12142427 PMC135234

[76] Corbishley A, Ahmad NI, Hughes K, Hutchings MR, McAteer SP, Connelley TK, et al Strain-dependent cellular immune responses in cattle following *Escherichia coli* O157:H7 colonization. Infection and immunity. 2014;82(12):5117–31. 10.1128/IAI.02462-14 25267838 PMC4249286

[77] Borten MA, Bajikar SS, Sasaki N, Clevers H, Janes KA. Automated brightfield morphometry of 3D organoid populations by OrganoSeg. Scientific reports. 2018;8(1):5319 10.1038/s41598-017-18815-8 29593296 PMC5871765

[78] Darling AE, Mau B, Perna NT. progressiveMauve: multiple genome alignment with gene gain, loss and rearrangement. PloS one. 2010;5(6):e11147 10.1371/journal.pone.0011147 20593022 PMC2892488

[79] Alikhan NF, Petty NK, Ben Zakour NL, Beatson SA. BLAST Ring Image Generator (BRIG): simple prokaryote genome comparisons. BMC genomics. 2011;12:402 10.1186/1471-2164-12-402 21824423 PMC3163573

[80] Stajich JE, Block D, Boulez K, Brenner SE, Chervitz SA, Dagdigian C, et al The Bioperl toolkit: Perl modules for the life sciences. Genome research. 2002;12(10):1611–8. 10.1101/gr.361602 12368254 PMC187536

[81] Page AJ, Cummins CA, Hunt M, Wong VK, Reuter S, Holden MT, et al Roary: rapid large-scale prokaryote pan genome analysis. Bioinformatics. 2015;31(22):3691–3. 10.1093/bioinformatics/btv421 26198102 PMC4817141

[82] Benjamini Y, Hochberg Y. Controlling the False Discovery Rate—a Practical and Powerful Approach to Multiple Testing. J Roy Stat Soc B Met. 1995;57(1):289–300.

[83] Team RC. R: A language and environment for statistical computing. R foundation for statistical computing, Vienna, Austria 2014.

