# Supplementary figures and images for: Shiga toxin sub-type 2a increases the efficiency of Escherichia coli O157 transmission between animals and restricts epithelial regeneration in bovine enteroids
Source: PLoS Pathog. 2019 Oct 3;15(10):e1008003. doi: 10.1371/journal.ppat.1008003 (PMC6776261; doi:10.1371/journal.ppat.1008003)

**A**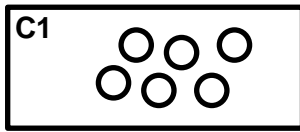**B**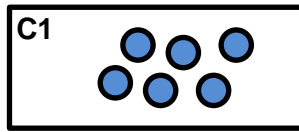**C**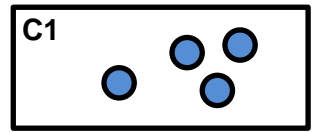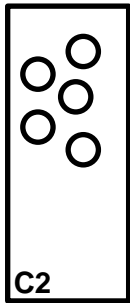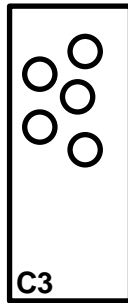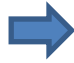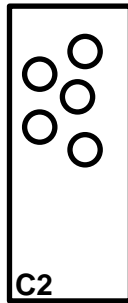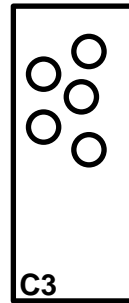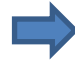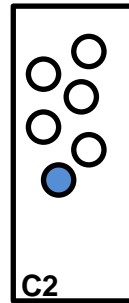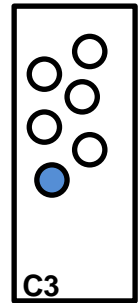

○ Naive uninfected calf    ● Experimentally infected calf

Supplement: S3 Fig — (A) Uninfected naïve calves (n = 5 per room) were housed in rooms C1, C2 and C3. (B) On day 0 all calves in room C1 were experimentally infected with ~109 CFU of E. coli O157 by orogastric intubation. (C) At five days post-challenge a calf shedding >104 cfu/g faeces was moved into rooms C2 and C3, respectively. Faecal bacterial shedding (cfu/g) and environmental levels were monitored daily for a further 18-day period. (PDF) [file ppat.1008003.s003.pdf]

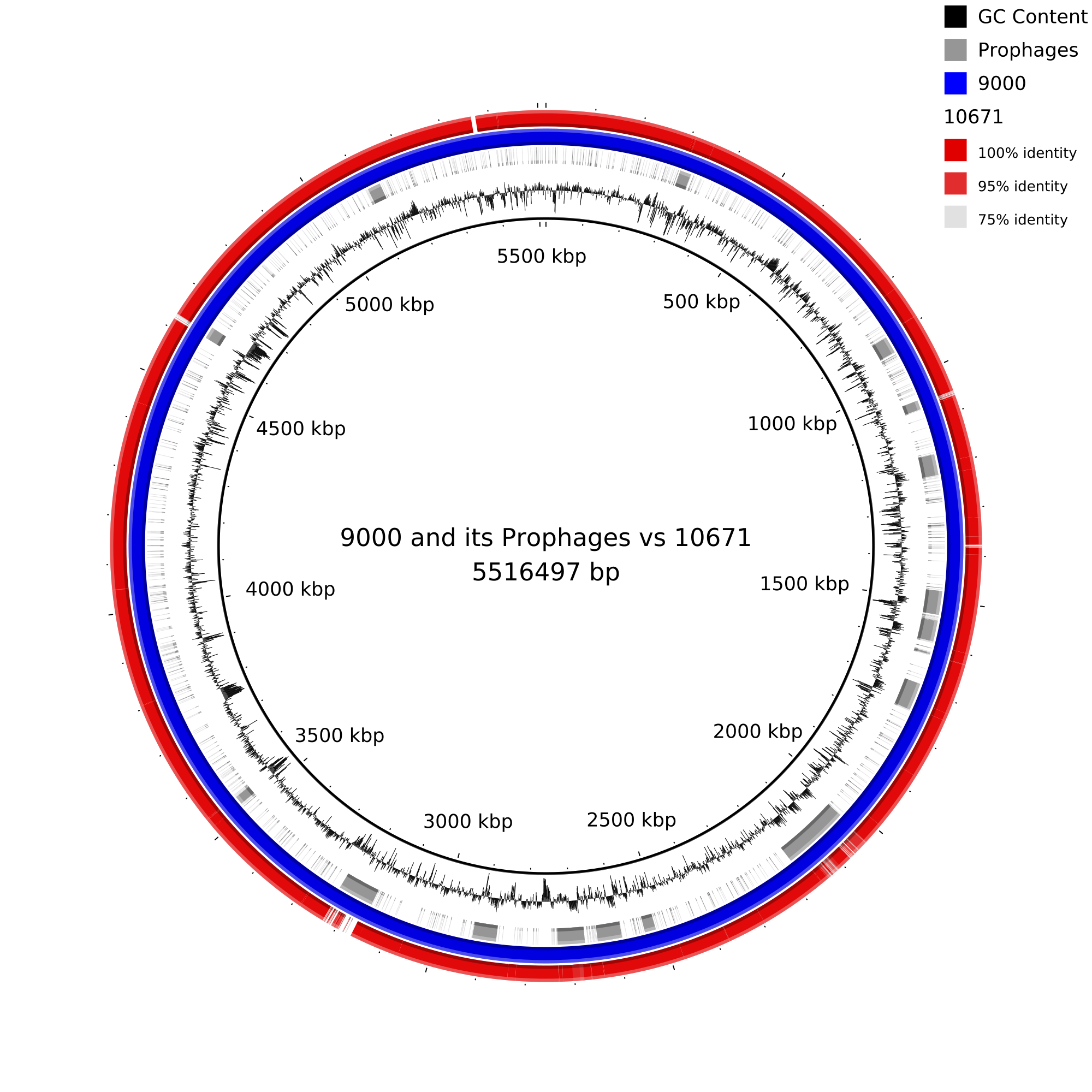

Supplement: S6 Fig — The genome of PT32 strain 10671 (red) was compared against reference PT21/28 strain 9000 (blue) for gene presence/absence. Annotated prophage (grey) and their loci, including Stx2aΦ centred at 3,200 kbp, are shown for strain 9000. (TIF) [file ppat.1008003.s006.tif]
