# Supplementary material for: Shiga toxin sub-type 2a increases the efficiency of Escherichia coli O157 transmission between animals and restricts epithelial regeneration in bovine enteroids
Source: PLoS Pathog. 2019 Oct 3;15(10):e1008003. doi: 10.1371/journal.ppat.1008003 (PMC6776261; doi:10.1371/journal.ppat.1008003)
Supplement: S5 Fig — Serum levels of (A) H7-specific; (B) Tir-specific; (C) EspA-specific and (D) Intimin-specific serum antibody levels in E. coli O157 challenged and unchallenged control calves. Levels of antigen-specific IgA, IgG1 and IgG2 in weekly serum samples collected from calves orally challenged with ~109 CFU E. coli O157 strains 9000, 9000R or 10671, or from unchallenged control calves were determined by indirect ELISA. Data represents the mean value ± SEM. (PDF) [file ppat.1008003.s005.pdf]

S5 Fig. Weekly serum antibody responses to strains 9000, 9000R and 10671.
Serum levels of (A) H7-specific; (B) Tir-specific; (C) EspA-specific and (D) Intimin-specific serum antibody levels in *E. coli* O157 challenged and unchallenged control calves. Levels of antigen-specific IgA, IgG1 and IgG2 in weekly serum samples collected from calves orally challenged with ~10^9 CFU of *E. coli* O157 strains 9000, 9000R or 10671, or from unchallenged control calves were determined by indirect ELISA. Data represents the mean value ± SEM.

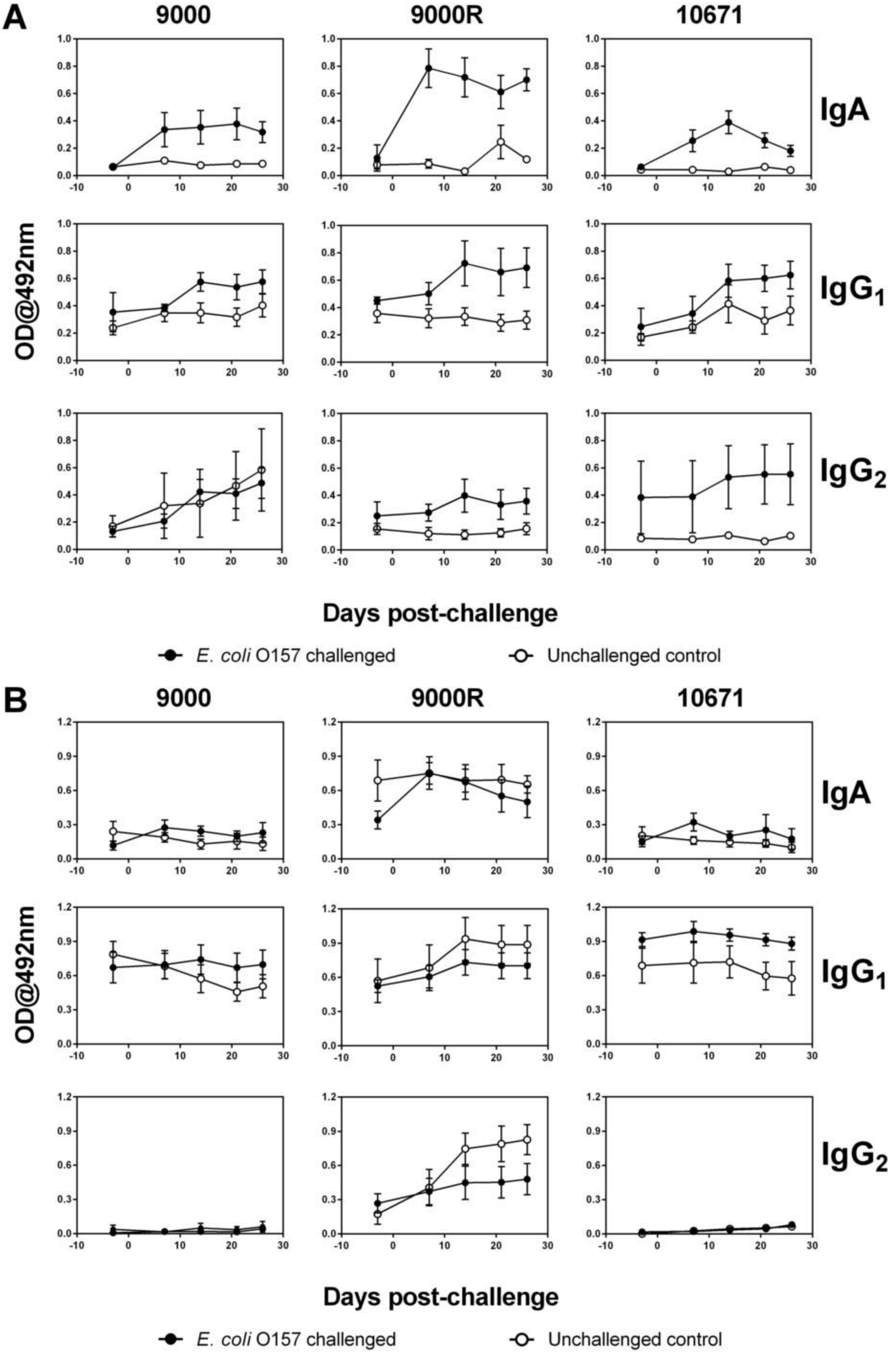

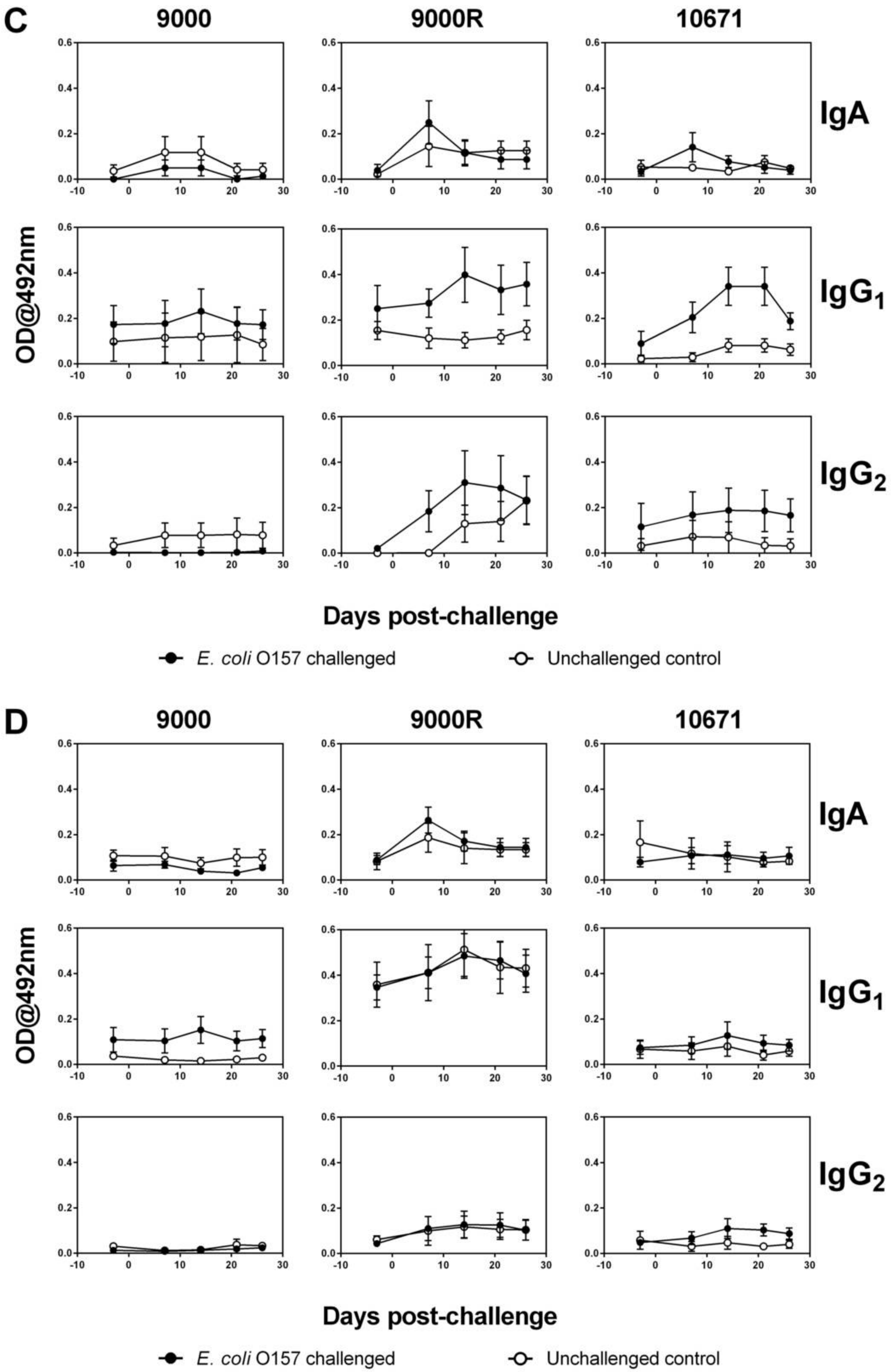

C
9000
9000R
10671
IgA
IgG$_1$
IgG$_2$
OD@492nm
Days post-challenge
E. coli O157 challenged
Unchallenged control
D
9000
9000R
10671
IgA
IgG$_1$
IgG$_2$
OD@492nm
Days post-challenge
E. coli O157 challenged
Unchallenged control
