# Supplementary material for: Shiga toxin sub-type 2a increases the efficiency of Escherichia coli O157 transmission between animals and restricts epithelial regeneration in bovine enteroids
Source: PLoS Pathog. 2019 Oct 3;15(10):e1008003. doi: 10.1371/journal.ppat.1008003 (PMC6776261; doi:10.1371/journal.ppat.1008003)
Supplement: S2 Table — (DOCX) [file ppat.1008003.s008.docx]

| **Name** | **Restriction Site** | **Sequence^a^** |
| --- | --- | --- |
| **Deletion of ISEc8^b^** |  |  |
| No stx2a |  | GTTTACGATCGTAAAAATCTGC |
| Ni stx2a | *Not*I | cgctctt gcggccgc TTGGAACGGATACAGGTGTTCCTTTTGGCTG |
| Co stx2a |  | TGTCGAATGTATCCGGCAATGG |
| Ci stx2a | *Not*I | ccgttccaa gcggccgc AAGAGCGGCATAACCTGATTCGTGGTATG |
| NiOE stx2a |  | CAGTGCCTGACGAAATTCTCTCTGTATCTGCCTGAAGCGTAAGGCTTC |
| CiOE stx2a |  | GAAGCCTTACGCTTCAGGCAGATACAGAGAGAATTTCGTCAGGCACTG |
| **Phage Curing** |  |  |
| Nt-pTOF24-TcR | *Pst*I | aaa ctgcag AGATCTTAACGCAGTCAGGCACCGTGTATG |
| Ct-pTOF24-TcR | *Xho*I | aaa ctcgag CGAGGTGCCGCCGGCTTCCATTCA |
| 5'-Sp5pL-PstI-IF | *Pst*I | gtctcggtacccgac ctgcag CCTCTCGCCCAAAAAAACACATAAC |
| 3'-Sp5pL-BglII-IF | *Bgl*II | tgcctgactgcgtta agatct TGCCAGTCTGTTCCATTTGGCTTCC |
| 5'-560stx2pL-PstI-IF | *Pst*I | gtctcggtacccgac ctgcag CTTTGCCTCACGTTCGCCCACC |
| 3'-560stx2pL-BglII-IF | *Bgl*II | tgcctgactgcgtta agatct TCCTGCTGACGATGATAATAATG |
| **Faecal Screening^c^** |  |  |
| stx1.fw |  | TTTGTYACTGTSACAGCWGAAGCYTTACG |
| stx1.Rv |  | CCCCAGTTCARWGTRAGRTCMACRTC |
| stx1 probe |  | YAKYE-CTGGATGATCTCAGTGGGCGTTCTTATGTAA-BHQ1 |
| stx2.fw |  | TTTGTYACTGTSACAGCWGAAGCYTTACG |
| stx2.Rv |  | CCCCAGTTCARWGTRAGRTCMACRTC |
| stx2 probe |  | FAM-TCGTCAGGCACTGTCTGAAACTGCTCC-BHQ1 |
| rfb.fw |  | TTTCACACTTATTGGATGGTCTCAA |
| rfb.Rv |  | CGATGAGTTTATCTGCAAGGTGAT |
| rfb probe |  | RED-AGGACCGCAGAGGAAAGAGAGGAATTAAGG-BHQ2 |
| **Φ Deletion** |  |  |
| stx2a.fw |  | ACTGGAGCGATTTCATCTGG |
| stx2a.Rv |  | CTTACCGTCAATCTTGTAGC |
| stx2c.fw |  | ATGTTTTCCGCCGCAAAACC |
| stx2c.Rv |  | GAATACAGGTCTTCGGTACG |
| **RT-qPCR** |  |  |
| stx2.fw |  | GAAGAAGATGTTTATGGCGGTTT |
| stx2a.Rv |  | CCCGTCAACCTTCACTGTAA |
| stx2c.Rv |  | TCCGGCCACTTTTACTGTGA |
| cro2a.fw |  | AGCGAAAGGGCTGTCTATAAG |
| cro2a.Rv |  | GCTTGATATTTGCCACCAGAAA |
| N2a.fw |  | GAAGTATTACACCGCCCTACTC |
| N2a.Rv |  | CGTCCTGTGCCATGAACTTA |
| cro2c.fw |  | CTATCGGACATTGGGAATCTGG |
| cro2c.Rv |  | GGGAACACGTCATCAAGACTTA |
| N2c.fw |  | GCAAAGTCAGGAGGCGATTA |
| N2c.Rv |  | CTTCTGCTCTCATTGCTGGT |
| ^a^ Restriction sites are underlined; Template sequences are in uppercase  ^b^ C and N denote 5' and 3' ends of PCR products respectively; i and o denote inner (outward-pointing) and outer (inward-pointing) primer; OE: primers used for overlap-extension  ^c^ YAKYE = Yakamina Yellow; FAM = 6-Carboxyfluorescein; Red = Texas Red | | |

**Table S2: Details of PCR primers used in this study**
